# A single-nucleus and spatial transcriptomic atlas of the COVID-19 liver reveals topological, functional, and regenerative organ disruption in patients

**DOI:** 10.1186/s13059-025-03499-5

**Published:** 2025-03-14

**Authors:** Yered Pita-Juarez, Dimitra Karagkouni, Nikolaos Kalavros, Johannes C. Melms, Sebastian Niezen, Toni M. Delorey, Adam L. Essene, Olga R. Brook, Deepti Pant, Disha Skelton-Badlani, Pourya Naderi, Pinzhu Huang, Liuliu Pan, Tyler Hether, Tallulah S. Andrews, Carly G. K. Ziegler, Jason Reeves, Andriy Myloserdnyy, Rachel Chen, Andy Nam, Stefan Phelan, Yan Liang, Mark Gregory, Shanshan He, Michael Patrick, Tushar Rane, Aster Wardhani, Amit Dipak Amin, Jana Biermann, Hanina Hibshoosh, Molly Veregge, Zachary Kramer, Christopher Jacobs, Yusuf Yalcin, Devan Phillips, Michal Slyper, Ayshwarya Subramanian, Orr Ashenberg, Zohar Bloom-Ackermann, Victoria M. Tran, James Gomez, Alexander Sturm, Shuting Zhang, Stephen J. Fleming, Sarah Warren, Joseph Beechem, Deborah Hung, Mehrtash Babadi, Robert F. Padera, Sonya A. MacParland, Gary D. Bader, Nasser Imad, Isaac H. Solomon, Eric Miller, Stefan Riedel, Caroline B. M. Porter, Alexandra-Chloé Villani, Linus T.-Y. Tsai, Winston Hide, Gyongyi Szabo, Jonathan Hecht, Orit Rozenblatt-Rosen, Alex K. Shalek, Benjamin Izar, Aviv Regev, Yury V. Popov, Z. Gordon Jiang, Ioannis S. Vlachos

**Affiliations:** 1 Department of Pathology, Beth Israel Deaconess Medical Center, Boston, MA USA; 2 Harvard Medical School, Boston, MA USA; 3 Broad Institute of MIT and Harvard, Cambridge, MA USA; 4 Spatial Technologies Unit, HMS Initiative for RNA Medicine / Beth Israel Deaconess Medical Center, Boston, MA USA; 5 Department of Medicine, Division of Hematology/Oncology, Columbia University Irving Medical Center, New York, NY USA; 6 Columbia Center for Translational Immunology, New York, NY USA; 7 Department of Medicine, Beth Israel Deaconess Medical Center, Boston, MA USA; 8 Division of Gastroenterology, Hepatology and Nutrition, Beth Israel Deaconess Medical Center, Boston, MA USA; 9 Klarman Cell Observatory, Broad Institute of MIT and Harvard, Cambridge, MA USA; 10 Division of Endocrinology, Diabetes, and Metabolism, Beth Israel Deaconess Medical Center, Boston, MA USA; 11 Boston Nutrition and Obesity Research Center Functional Genomics and Bioinformatics Core, Boston, MA USA; 12 Department of Radiology, Beth Israel Deaconess Medical Center, Boston, MA USA; 13 NanoString Technologies, Inc., Seattle, WA USA; 14 Ajmera Transplant Centre, Toronto General Research Institute, University Health Network, Toronto, ON Canada; 15 Program in Health Sciences & Technology, Harvard Medical School & Massachusetts Institute of Technology, Boston, MA USA; 16 Institute for Medical Engineering & Science, Massachusetts Institute of Technology, Cambridge, MA USA; 17 Koch Institute for Integrative Cancer Research, Massachusetts Institute of Technology, Cambridge, MA USA; 18 Ragon Institute of MGH, MIT, and Harvard, Cambridge, MA USA; 19 Harvard Graduate Program in Biophysics, Harvard University, Cambridge, MA USA; 20 Harvard Stem Cell Institute, Cambridge, MA USA; 21 Program in Computational & Systems Biology, Massachusetts Institute of Technology, Cambridge, MA USA; 22 Program in Immunology, Harvard Medical School, Boston, MA USA; 23 Department of Chemistry, Massachusetts Institute of Technology, Cambridge, MA USA; 24 Department of Pathology and Cell Biology, Columbia University Irving Medical Center, New York, NY USA; 25 Present Address: Genentech, 1 DNA Way, South San Francisco, CA USA; 26 Infectious Disease and Microbiome Program, Broad Institute of MIT and Harvard, Cambridge, MA USA; 27 Data Sciences Platform, Broad Institute of MIT and Harvard, Cambridge, MA USA; 28 Precision Cardiology Laboratory, Broad Institute of MIT and Harvard, Cambridge, MA USA; 29 Department of Genetics, Harvard Medical School, Boston, MA USA; 30 Department of Molecular Biology and Center for Computational and Integrative Biology, Massachusetts General Hospital, Boston, MA USA; 31 Department of Pathology, Brigham and Women’s Hospital, Boston, MA USA; 32 Department of Immunology, University of Toronto, Medical Sciences Building, 1 King’s College Circle, Toronto, ON Canada; 33 Department of Laboratory Medicine and Pathobiology, University of Toronto, Toronto, ON Canada; 34 Department of Molecular Genetics, University of Toronto, Toronto, ON Canada; 35 The Donnelly Centre, Toronto, ON Canada; 36 Center for Immunology and Inflammatory Diseases, Department of Medicine, Massachusetts General Hospital, Boston, MA USA; 37 Center for Cancer Research, Massachusetts General Hospital, Harvard Medical School, Boston, MA USA; 38 Department of Medicine, Harvard Medical School, Boston, MA USA; 39 Herbert Irving Comprehensive Cancer Center, Columbia University Irving Medical Center, New York, NY USA; 40 Program for Mathematical Genomics, Columbia University Irving Medical Center, New York, NY USA; 41 Department of Systems Biology, Columbia University Irving Medical Center, New York, NY USA; 42 Cancer Research Institute, Beth Israel Deaconess Medical Center, Boston, MA USA; 43 Harvard Medical School Initiative for RNA Medicine, Boston, MA USA

**Keywords:** SARS-CoV-2, COVID-19, Spatial transcriptomics, Single-nucleus sequencing, Liver, Single-cell sequencing

## Abstract

**Background:**

The molecular underpinnings of organ dysfunction in severe COVID-19 and its potential long-term sequelae are under intense investigation. To shed light on these in the context of liver function, we perform single-nucleus RNA-seq and spatial transcriptomic profiling of livers from 17 COVID-19 decedents.

**Results:**

We identify hepatocytes positive for SARS-CoV-2 RNA with an expression phenotype resembling infected lung epithelial cells, and a central role in a pro-fibrotic TGFβ signaling cell–cell communications network. Integrated analysis and comparisons with healthy controls reveal extensive changes in the cellular composition and expression states in COVID-19 liver, providing the underpinning of hepatocellular injury, ductular reaction, pathologic vascular expansion, and fibrogenesis characteristic of COVID-19 cholangiopathy. We also observe Kupffer cell proliferation and erythrocyte progenitors for the first time in a human liver single-cell atlas. Despite the absence of a clinical acute liver injury phenotype, endothelial cell composition is dramatically impacted in COVID-19, concomitantly with extensive alterations and profibrogenic activation of reactive cholangiocytes and mesenchymal cells.

**Conclusions:**

Our atlas provides novel insights into liver physiology and pathology in COVID-19 and forms a foundational resource for its investigation and understanding.

**Supplementary Information:**

The online version contains supplementary material available at 10.1186/s13059-025-03499-5.

## Background

COVID-19 exhibits a wide phenotypic spectrum with potential multi-organ involvement during its acute phase [1], including liver-related pathology. Abnormal liver biochemistry is reported in 15–65% of SARS-CoV-2 infected individuals [2–4] and is often associated with poorer clinical outcomes [3, 4]. To date, there are few studies of human liver tissue from COVID-19 patients, hindering in-depth investigations of COVID-19-related liver injury, its main causes, and potential long-term effects, especially post-acute sequelae of SARS-CoV-2 infection (PASC), such as the patient-coined term “long COVID” [5] and post-COVID cholangiopathy, an emerging entity that may require liver transplantation [6]. In our previous work [7, 8], we assembled a multi-tissue COVID-19 cell atlas across lung, heart, kidney, and liver, collected at autopsy from patients who succumbed to the disease and captured both parenchymal and non-parenchymal cell populations in epithelial tissues at high fidelity with single-nucleus RNA-seq (snRNA-seq). While we have investigated the COVID-19 pathobiology of the acute respiratory distress syndrome (ARDS) lung in depth, including by spatial -omics in situ, the impact in other organs, including the liver, have not yet been deeply explored.

Multiple factors may underlie the COVID-19 liver phenotype, including the impact of direct infection given the expression of SARS-CoV-2 entry factors in major hepatic cell classes [3, 9, 10], systemic inflammation, drug-induced injury, and hypoxia [3, 11]. Some studies suggest the presence of subclinical liver damage, especially in the liver vasculature [12], with short- and potentially long-term implications.

Metabolic, vascular, and biliary alterations in COVID-19 patients could result from direct or indirect viral damage to the liver [3], while it was recently shown through bulk RNA sequencing and proteomics that bulk gene and protein profiles of livers identified as positive with SARS-CoV-2 present similarities to the signatures associated with multiple other viral infections of the human liver [4]. This further increases the importance of identifying its effects on infected cells and their interactions with their microenvironment. The spatial manifestation of COVID-19 phenotypes in the liver could especially be of interest due to its distinct architecture. The liver is organized in the hexagonal-shaped repeating anatomical units of the liver lobules, radiating into spatially distinct lobular zones that span from the portal triad to the central vein. The oxygen and nutrition gradients between the portal and central vein dictate liver development and define cellular function. While cellular expression programs are affected by zonation in both health and disease [13, 14], most spatial and zonation information to date has been derived from selected markers or by concordance with animal models [13].

Here, we created an integrated liver COVID-19 atlas of 80,808 snRNA-seq profiles from liver samples collected at autopsy from 17 patients who succumbed to severe COVID-19, as well as whole transcriptome spatial profiling of 62 regions of interest (ROIs) from four concordant livers. By comparison with healthy controls (*n* = 4), we generated a high-resolution map of the cellular landscape of the COVID-19 liver as well as determination of the viral impact on cell subsets, their activation states, and cell–cell communication. We used these to assess clinically relevant changes in hepatocytes and hepatic non-parenchymal cells in response to viral infection.

## Results

### A liver cell and spatial atlas in severe COVID-19

**Fig. 1:**
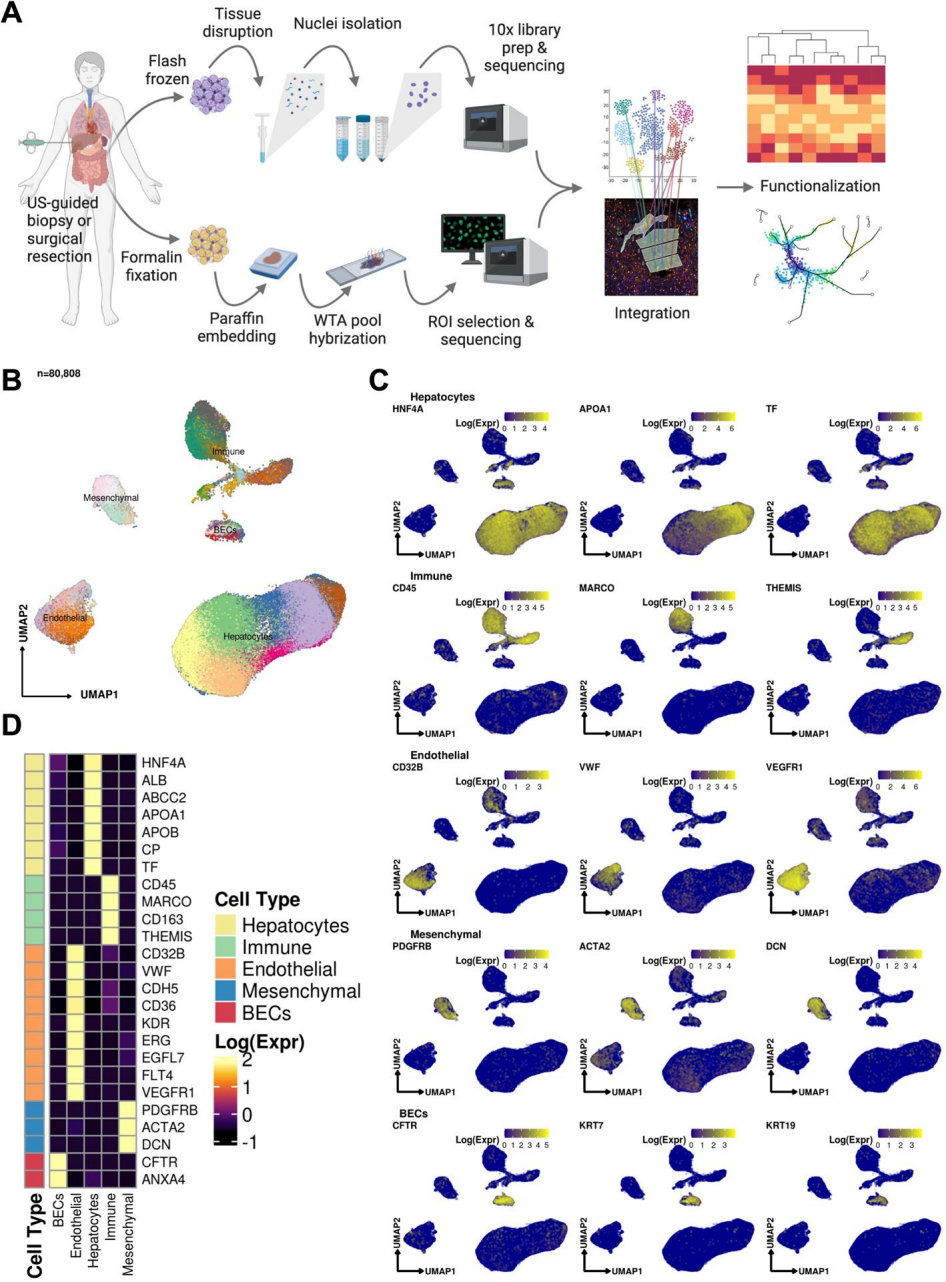
**A** Sample processing pipeline depicting sample acquisition, preparation for snRNAseq and spatial transcriptomic profiling, data generation, integration, and in silico functionalization. **B** Uniform manifold approximation and projection (UMAP) for all cells passing quality control (*n* = 80,808, Hepatocytes, *n* = 51,605; Immune / blood cells, *n* = 12,346; Endothelial cells: *n* = 9278, Mesenchymal cells (*n* = 4647); Biliary epithelial cells / Cholangiocytes, *n* = 2932). **C** Heatmap capturing the expression of marker genes across the 5 major compartments. **D** UMAP plots depicting gene marker expression for each compartment

To construct a COVID-19 liver atlas, we leveraged an autopsy cohort of 17 COVID-19 patients (6 males, 11 females, ages from 30–35 to > 89 years) across four medical centers from the Northeastern United States (Table 1, Fig. 1a) [7, 8, 15]. All samples were obtained postmortem using either ultrasound-guided needle biopsy or surgical dissection by following stringent protocols established previously [7] (Additional file 1: Table S1, Methods). Most patients had multiorgan failure at the time of death. While liver function serum markers within 24 h of death showed varying degrees of transaminitis, no patient had clinical or laboratory signs of liver failure or ongoing liver injury (Additional file 1: Table S2).

**Table 1:** COVID-19 and control cohort overview. (PMI: post-mortem interval)

**Cohort**	**Donor**	**Race**	**Ethnicity**	**Sex**	**Age**	**Organ failure**	**snRNAseq**	**DSP WTA**	**BMI**	**Pre-existing condition**	**Pre-existing heart disease**	**Pre-existing lung disease**
A	L1	Black/AA	Not Hispanic/Latino	M	80-89	Lung	x	x	18.5-24.9 (normal)	Asthma, COPD, Diabetes, Myocardial infarction, Kidney failure, Hypertension, Immunocompromised	No	No
A	L2	Black/AA	Not Hispanic/Latino	F	80-89	Lung	x	x	30.0-39.9 (obese)	Asthma, COPD, Diabetes, Myocardial infarction, Kidney failure, Hypertension, Immunocompromised	Coronary artery disease, congestive heart failure, valvular disease	Asthma, COPD, requiring home O2, any chronic lung condition
A	L3	Other	Unknown/not documented	F	50-59	Lung, Kidney	x	x	Unknown	Asthma, COPD, Diabetes, Myocardial infarction, Kidney failure, Hypertension, Immunocompromised	No	Asthma, COPD, requiring home O2, any chronic lung condition
A	L4	Black/AA	Not Hispanic/Latino	F	70-79	Lung, Heart	x	x	30.0-39.9 (obese)	Asthma, COPD, Diabetes, Myocardial infarction, Kidney failure, Hypertension, Immunocompromised	No	No
A	L5	White	Not Hispanic/Latino	M	70-79	Lung, Kidney, Heart	x		Unknown	Asthma, COPD, Diabetes, Myocardial infarction, Kidney failure, Hypertension, Immunocompromised	No	No
B	L6	Other	Hispanic/Latino	F	60-69	Multi-organ	x		30.0-39.9 (obese)	Asthma, COPD, Diabetes, Myocardial infarction, Kidney failure, Hypertension, Immunocompromised	No	No
B	L7	Other	Hispanic/Latino	M	60-69	Multi-organ	x		30.0-39.9 (obese)	Asthma, COPD, Diabetes, Myocardial infarction, Kidney failure, Hypertension, Immunocompromised	Coronary artery disease, congestive heart failure, valvular disease	Asthma, COPD, requiring home O2, any chronic lung condition
B	L8	Black/AA	Not Hispanic/Latino	M	30-39	Lung, heart	x		25.0-29.9 (overweight)	Asthma, COPD, Diabetes, Myocardial infarction, Kidney failure, Hypertension, Immunocompromised	No	No
B	L9	Other	Hispanic/Latino	M	40-49	Lung, kidney	x		>=40 (severely obese)	No	No	No
B	L10	Black/AA	Not Hispanic/Latino	F	80-89	Lung	x		25.0-29.9 (overweight)	Asthma, COPD, Diabetes, Myocardial infarction, Kidney failure, Hypertension, Immunocompromised	Coronary artery disease, congestive heart failure, valvular disease	Asthma, COPD, requiring home O2, any chronic lung condition
B	L11	White	Not Hispanic/Latino	M	80-89	Lung	x		18.5-24.9 (normal)	Asthma, COPD, Diabetes, Myocardial infarction, Kidney failure, Hypertension, Immunocompromised	Coronary artery disease, congestive heart failure, valvular disease	Asthma, COPD, requiring home O2, any chronic lung condition
C	L12	White	Not Hispanic/Latino	M	50-59	Multi-organ	x		30.0-39.9 (obese)	No	No	No
C	L13	White	Not Hispanic/Latino	M	70-79	lung and kidney	x		30.0-39.9 (obese)	Asthma, COPD, Diabetes, Myocardial infarction, Kidney failure, Hypertension, Immunocompromised	Coronary artery disease, congestive heart failure, valvular disease	No
D	L14	White	Not Hispanic/Latino	M	70-79	Heart	x		25.0-29.9 (overweight)	Asthma, COPD, Diabetes, Myocardial infarction, Kidney failure, Hypertension, Immunocompromised	Coronary artery disease, congestive heart failure, valvular disease	Asthma, COPD, requiring home O2, any chronic lung condition
D	L15	Unknown	Hispanic/Latino	F	80-89	Lung	x		30.0-39.9 (obese)	Asthma, COPD, Diabetes, Myocardial infarction, Kidney failure, Hypertension, Immunocompromised	No	No
D	L16	Unknown	Hispanic/Latino	M	70-79	Lung	x		Unknown	Asthma, COPD, Diabetes, Myocardial infarction, Kidney failure, Hypertension, Immunocompromised	Coronary artery disease, congestive heart failure, valvular disease	No
D	L17	Unknown	Hispanic/Latino	M	80-89	Lung	x		18.5-24.9 (normal)	Asthma, COPD, Diabetes, Myocardial infarction, Kidney failure, Hypertension, Immunocompromised	No	No
**Controls**												
E	L18	NA	NA	F	NA	NA	x					
E	L19	NA	NA	M	NA	NA	x					
E	L20	NA	NA	F	NA	NA	x					
E	L21	NA	NA	F	NA	NA	x					

**Cohort**	**Donor**	**Pre-existing kidney disease**	**Pre-existing diabetes**	**Pre-existing hypertension**	**Pre-existing immunocompromised condition**	**Smoking status**	**Respiratory symptoms**	**Intermittent mandatory ventilation (IMV) (days)**	**Symptoms to death (days)**	**Post-mortem interval**
A	L1	chronic kidney disease, baseline creatinine >1.5, ESRD)	No	No	No	Never or unknown	Sore throat, congestion, productive or dry cough, shortness of breath or hypoxia, or chest pain)	0	11	1 h
A	L2	No	Pre-diabetes, insulin and non-insulin dependent diabetes	Yes	No	Prior	Sore throat, congestion, productive or dry cough, shortness of breath or hypoxia, or chest pain)	0	17	3 h 4 min
A	L3	No	Pre-diabetes, insulin and non-insulin dependent diabetes	Yes	No	Never or unknown	Sore throat, congestion, productive or dry cough, shortness of breath or hypoxia, or chest pain)	30	41	2 h 43 min
A	L4	chronic kidney disease, baseline creatinine >1.5, ESRD)	No	Yes	No	Prior	Sore throat, congestion, productive or dry cough, shortness of breath or hypoxia, or chest pain)	13	16	1 h 30 min
A	L5	No	Pre-diabetes, insulin and non-insulin dependent diabetes	Yes	No	Never or unknown	No	24	31	1 h 25 min
B	L6	No	Pre-diabetes, insulin and non-insulin dependent diabetes	Yes	No	Never or unknown	Sore throat, congestion, productive or dry cough, shortness of breath or hypoxia, or chest pain)	12	18	18 h
B	L7	No	Pre-diabetes, insulin and non-insulin dependent diabetes	Yes	No	Never or unknown	Sore throat, congestion, productive or dry cough, shortness of breath or hypoxia, or chest pain)	7	12	20 h
B	L8	No	Pre-diabetes, insulin and non-insulin dependent diabetes	Yes	No	Never or unknown	Sore throat, congestion, productive or dry cough, shortness of breath or hypoxia, or chest pain)	6	6	16 h
B	L9	No	No	No	No	Never or unknown	Sore throat, congestion, productive or dry cough, shortness of breath or hypoxia, or chest pain)	9	22	15 h
B	L10	No	No	Yes	No	Never or unknown	Sore throat, congestion, productive or dry cough, shortness of breath or hypoxia, or chest pain)	0	19	10 h
B	L11	No	No	Yes	No	Prior	Sore throat, congestion, productive or dry cough, shortness of breath or hypoxia, or chest pain)	0	9	17 h
C	L12	No	No	No	No	Current	Sore throat, congestion, productive or dry cough, shortness of breath or hypoxia, or chest pain)	4	8	20 h
C	L13	No	Pre-diabetes, insulin and non-insulin dependent diabetes	Yes	No	Prior	Sore throat, congestion, productive or dry cough, shortness of breath or hypoxia, or chest pain)	4	7	24 h
D	L14	chronic kidney disease, baseline creatinine >1.5, ESRD)	Pre-diabetes, insulin and non-insulin dependent diabetes	Yes	No	Prior	Sore throat, congestion, productive or dry cough, shortness of breath or hypoxia, or chest pain)	0	16	4 h
D	L15	No	Pre-diabetes, insulin and non-insulin dependent diabetes	Yes	No	Prior	Sore throat, congestion, productive or dry cough, shortness of breath or hypoxia, or chest pain)	25	27	4 h
D	L16	No	No	Yes	No	Never or unknown	Sore throat, congestion, productive or dry cough, shortness of breath or hypoxia, or chest pain)	1	4	4 h 30 min
D	L17	No	No	Yes	No	Never or unknown	Sore throat, congestion, productive or dry cough, shortness of breath or hypoxia, or chest pain)	0	10	3 h 30 min
**Control**										
E	L18									
E	L19									
E	L20									
E	L21

We used snRNA-seq to collect 80,808 high-quality profiles from 17 COVID-19 patient autopsies (Methods) and integrated them computationally with snRNA-seq profiles from four healthy controls, prepared using a comparable protocol [16]. Following ambient RNA removal, quality control (QC), and preprocessing (Methods), we implemented a batch correction pipeline to generate corrected unique molecular identifier (UMI) counts per cell [17–19], which facilitated marker detection and cell type identification (Methods). The COVID-19 nucleus profiles were partitioned into five major compartments: hepatocytes (*k* = 51,605 cells; 63.8% of all nuclei); immune/blood (*k* = 12,346; 15.3%); endothelial (*k* = 9278; 11.5%), mesenchymal (*k* = 4647; 5.8%), and biliary epithelial cells (BECs) (*k* = 2932; 3.6%) (Fig. 1b–d, Additional file 2: Fig. S1a,b), spanning 50 cell subsets in distinct clusters (Additional file 3: Cluster Dictionary).

**Fig. 2:**
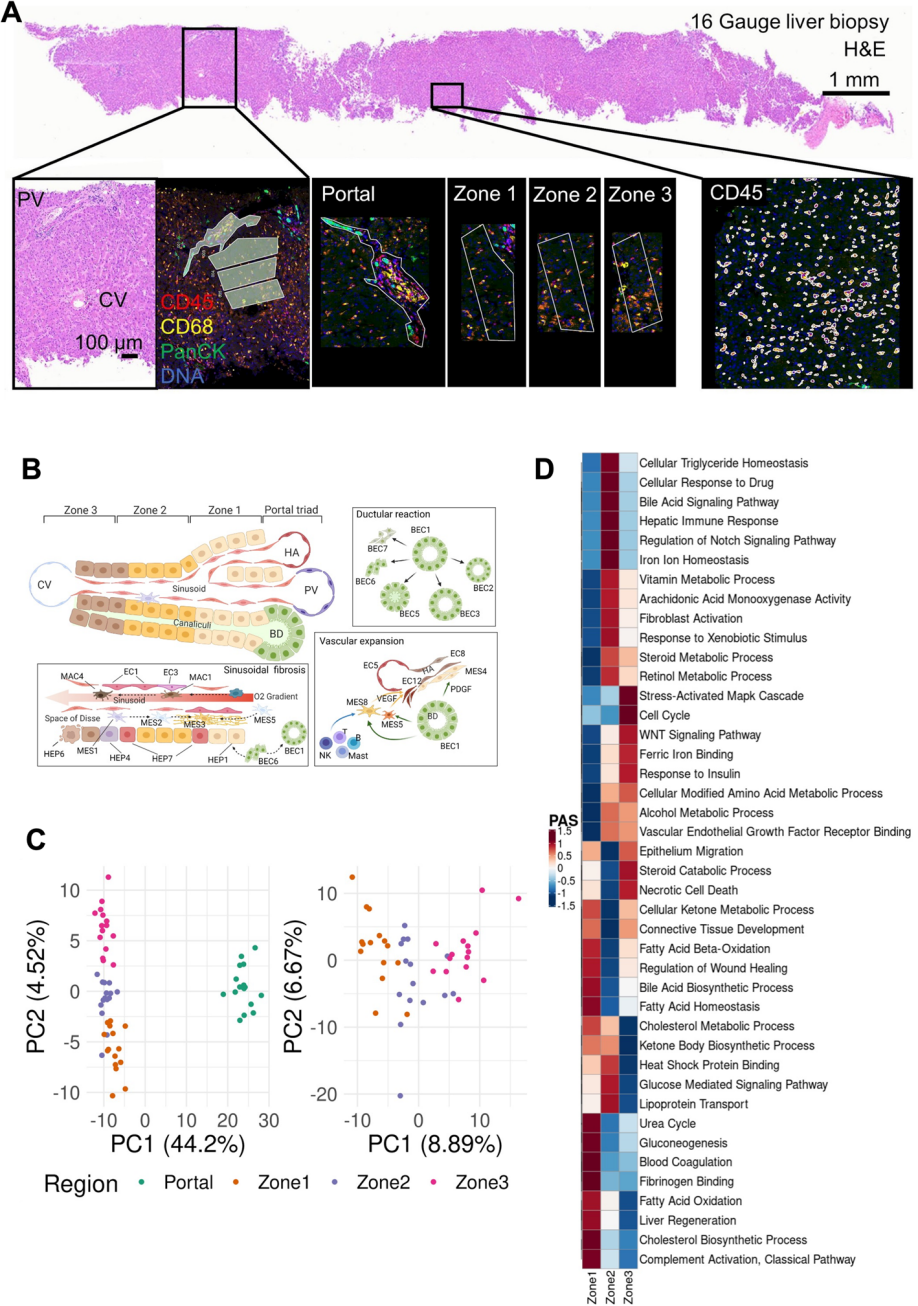
Overview of the digital spatial profiling. **A** Regions of interest (ROIs), corresponding to the liver lobule and the portal area. Gene expression in each region was profiled using the NanoString GeoMx Digital Spatial Profiling (DSP) Whole Transcriptome Atlas (WTA) platform. **B** Diagram of the spatial arrangement of cellular subpopulations in the liver lobule and interactions in the context of COVID-19 (HA, hepatic artery; PV, portal vein; CV, central vein; BD, bile duct). **C** Principal component analysis (PCA) embeddings based on batch-corrected probe counts of all ROIs (right) and for the liver lobule ROIs (left) reveal that the DSP WTA platform correctly separates the lobular region from the portal, and reveals significant expression differences between the 3 zones. **D** Normalized pathway activity scores (PAS) between lobule regions. The DSP WTA is able to capture known zone-specific pathways as well as reveal perturbed pathways related to liver pathology and viral infection

In parallel, we generated a spatial transcriptomic atlas from 62 Regions of Interest (ROIs) from lobular zones 1, 2, and 3, and the portal triad across four patient autopsies using the NanoString GeoMx Digital Spatial Profiling (DSP) Whole Transcriptome Atlas (WTA) platform (Fig. 1a, Methods). We first performed multiplexed immunofluorescence (Pan-cytokeratin (PanCK), CD45, CD68, Syto 83) on the same slides to define the lobular structure by identifying the portal triad and central vein as landmarks, as well as RNA in situ hybridization (RNA ISH) performed on a serial section against *ACE2*, *TMPRSS2*, and SARS-CoV-2 RNA to take also into account localized viral presence (Fig. 2a, Additional file 2: Fig. S1c, Methods). We then selected 62 ROIs, corresponding to lobular zones 1, 2, and 3, and the portal triad, by the consensus opinion of an expert panel of pathologists (J.H., S.R.), hepatologists (Z.G.J., Y.P., G.S.), and technology specialists (L.P., Y.L., Y.P-J., L.T., I.S.V.). We captured the expression of over 18,300 genes on the WTA, including 27 SARS-CoV-2-relevant probes (Additional file 1: Table S2). We further developed and applied an optimized pipeline for NanoString DSP WTA data normalization and preprocessing (Methods). The snRNA-seq and spatial profiles were interpreted and integrated using batch-corrected markers, a streamlined method for assigning pathway activity scores (PAS) (Methods), and by spatial registration of snRNA-seq profiles and signatures to decipher the localized interactions of cell types in the context of liver architecture (Fig. 2b, Methods). Finally, to evaluate whether rare cell types identified in COVID-19 patient liver samples were present in healthy liver tissue, as well as their localization, we interrogated 306,524 cells from a control liver sample using 1000-plex single-cell resolution spatial transcriptomics assay with the NanoString CosMx platform (Methods).

### Distinct zonal expression programs and their alterations in the COVID-19 liver

Each of the spatial transcriptomic ROI classes—three lobular zones and the portal triad—exhibited distinct expression profiles, with differential engagement of hepatic cellular pathways across the liver lobule, demonstrating the expected zonal division of hepatocellular function in the healthy liver [13] as well as its alteration in COVID-19. Principal component analyses (PCA) of the spatially defined expression profiles captured expression segregation between the portal triad and all lobular zones as well as among the three lobular zonal ROIs 1, 2, and 3 (Fig. 2c). Each region class was characterized by the differential expression of distinct region-specific markers and of functional gene sets [13, 20–22] (Fig. 2d). Based on a pathway activity score (PAS) analysis (Fig. 2d, Additional file 2: Fig. S1d,e, Methods), Zone 1 exhibits high activity of transcriptional programs for lipid and glutathione metabolism, urea cycle, fatty acid and steroid biosynthesis, and lipoprotein assembly, all commonly associated with liver-specific functions. Zone 2 follows similar patterns, but with higher activity of triglyceride catabolism and fucose biosynthesis. In contrast, Zone 3 exhibited high activity of drug catabolism programs. These processes are concordant with our current functional understanding of the zonated liver and have implications for chronic liver diseases. For instance: (1) hepatic steatosis typically starts in Zone 3 [23] in metabolic dysfunction associated fatty liver disease (MAFLD) and alcohol-related liver disease likely due to the lower metabolic activity; (2) drug-induced liver injury is most significant in the pericentral area as a result of drug catabolism; (3) disease related to impaired metabolism may manifest preferentially in Zone 1; and (4) Zone 1 predilection of pediatric NAFLD may in part be driven by genetic variants impacting lipid and lipoprotein metabolism, such as *PNPLA3* [24].

In COVID-19, we found evidence of a spatially orchestrated COVID-19-specific liver phenotype, including hepatocyte proliferation in Zone 1 as well as hypoxia and stress response pathways in Zone 3, which has not been reported in healthy liver. The phenotype was reflected by high activity scores of specific pathways across liver zones and the portal triad (Additional file 1: Table S3, Table S4, Additional file 2: Fig. S1d). Nonparenchymal cells showed distinct zonation of cellular physiology in the COVID-19 liver. For instance, among endothelial expression programs, differentiation programs were strongest in portal ROIs, programs for regulation of endothelial barrier establishment were highest in Zone 1, and endothelial cell chemotaxis in Zone 2 (Additional file 2: Fig. S1d). Among immune cells, portal ROIs exhibited high activity of monocyte activation and differentiation, as well as lymphocyte differentiation, whereas Zone 1 was characterized by Kupffer cell (KC) and natural killer (NK) cell proliferation, and lymphocyte migration and activation (Additional file 2: Fig. S1d). Among mesenchymal cells, portal ROIs had the highest activity of fibrogenic hepatic stellate cell (myofibroblast) activation, including response to platelet-derived growth factor (*PDGF*), fibroblast growth factor receptor (*FGFR*), and collagen/extracellular matrix production and organization pathways. Finally, Zone 3 exhibited the highest inflammation signals, including inflammasome activation, signaling by interleukins, response to cytokines, interferon-gamma binding, and inflammatory cell apoptotic processes (Additional file 2: Fig. S1d), which may be associated with SARS-CoV-2 infection and are not expected to be pronounced in Zone 3 in healthy livers. Thus, Zone 3 seems to be most severely affected by COVID-19.

### A spectrum of hepatocyte subsets from progenitors to functionally mature cells suggest plasticity of liver cells during injury

**Fig. 3:**
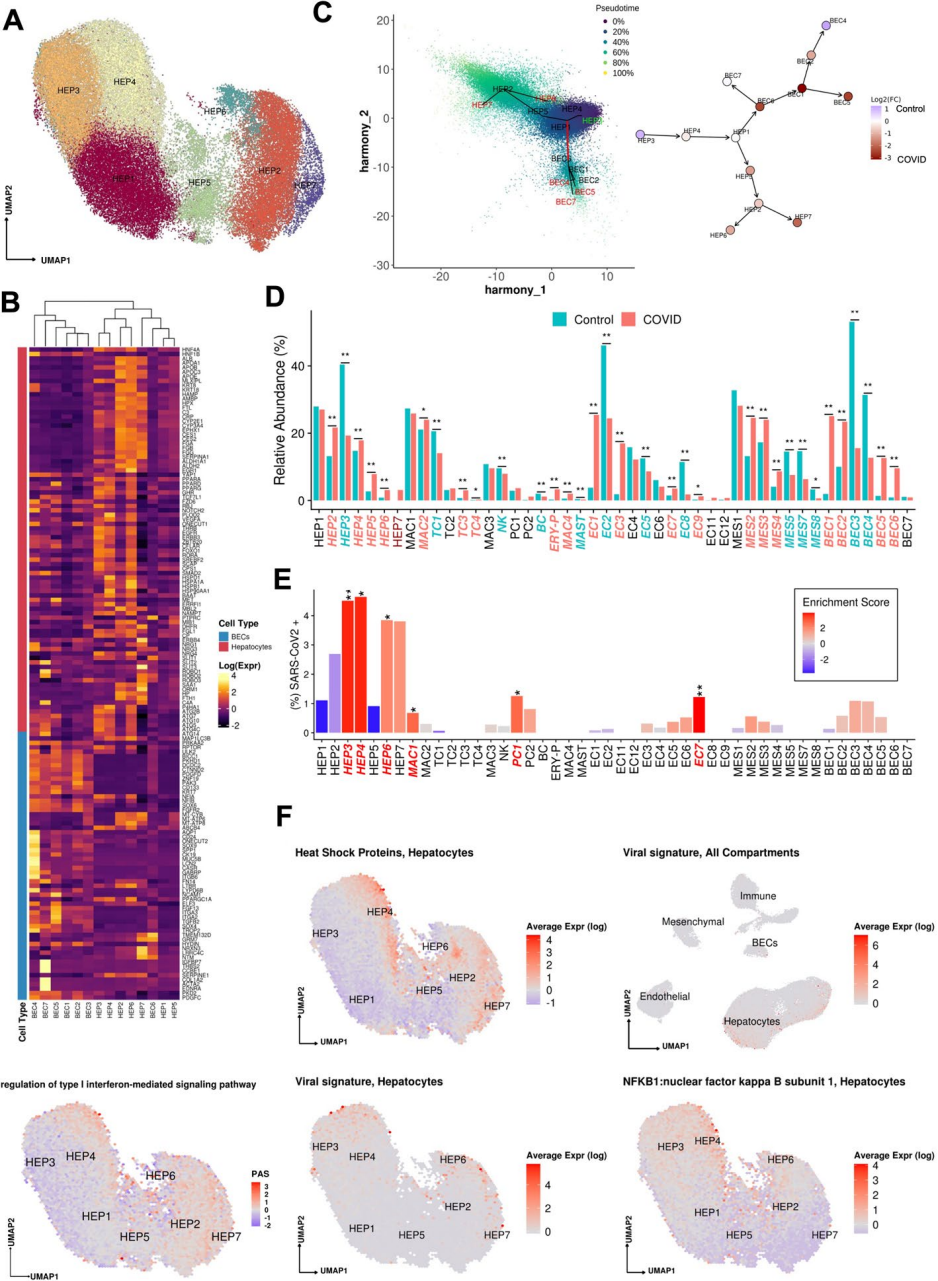
**A** Uniform manifold approximation and projection (UMAP) for Hepatocytes (HEP1 *n* = 13,951, HEP2 *n* = 11,187, HEP3 *n* = 9956, HEP4 *n* = 9241, HEP5 *n* = 4056, HEP6 *n* = 1612, HEP7 *n* = 1602). **B** Heatmap capturing the expression of marker genes across the hepatocyte and the biliary epithelial cell compartments. **C** Slingshot pseudotime values (left) projected on the 2 primary harmony embeddings across 5 lineages for hepatocyte and biliary epithelial cells from COVID-19 and healthy liver nuclei. The starting and ending lineage points are represented with green and red, respectively. Slingshot-derived lineages (right), coupled with cell composition fold-change differences between healthy and COVID-19 liver samples on a log2 scale. **D** Cell proportion differences between COVID-19 and healthy liver samples. Significantly different proportions are marked in red (higher in COVID-19), in blue (higher in Controls), and denoted with * (* FDR < 0.05, ** FDR < 0.01; Binomial Generalized Linear Mixed Model). COVID-19-specific clusters are denoted with dark red. **E** Abundance of SARS-CoV-2 RNA+ nuclei in the snRNAseq clusters. The bars are colored by the scaled viral enrichment score estimated per cluster. Significantly enriched clusters are marked in red and denoted with * (* FDR < 0.05, ** FDR < 0.01; Viral enrichment test). **F** Uniform manifold approximation and projection (UMAP) plots depicting the average expression of different heat shock proteins (*HSPA1A, HSPA1B, HSPA5, HSPA6, HSPA9, HSPB1, HSPD1*) in hepatocytes (upper left), pathway activity scores for GO term “regulation of type I interferon-mediated signaling pathway” (GO:0060338, bottom left), the viral load in all the cellular compartments (upper right), in hepatocytes (lower middle), and the average expression on *NFKB1* in hepatocytes (lower right)

Hepatocytes were the most populous compartment in the COVID-19 snRNA-seq atlas (63.8%) (Fig. 3a, Additional file 3: Cluster Dictionary) thanks to the ability of single-nucleus sequencing to capture this often underrepresented cell type in single-cell assays. Hepatocytes partitioned into seven subsets that spanned a continuum between two dichotomous ends: (1) primary essential liver functions, such as production of blood proteins, and (2) cell differentiation and replenishment, along with response to stress (Fig. 3a, Additional file 2: Fig. S2a,b). Regarding liver function, HEP2 cells (21.7% of hepatocytes) highly expressed genes encoding circulating blood proteins, including albumin, coagulation factors, and apolipoproteins (Fig. 3b), suggesting that only a fraction of all hepatocytes carry out conventional essential liver functions. HEP6 and HEP7 cells had similar profiles to those in the HEP2 subset but with high expression of acute phase proteins in HEP7 (e.g., *CRP*, *C3*, *C4a*, *SAA1*, and *FTH1*; a COVID-19 specific cluster; below) or apoptosis and cellular senescence pathways in HEP6 (Fig. 3b, Additional file 2: Fig. S3a). In contrast, cells in the HEP1, HEP3, and HEP4 subsets (Fig. 3a) exhibited lower levels of liver metabolic or synthetic function genes, but higher levels of cellular differentiation, wound healing, and signal transduction pathways (Additional file 2: Fig. S3a,b), such as the *HNF4A*/*HNF4B*, *YAP*/*TAZ*, *PPRA*/*B*/*G*, and GHR signaling pathways. HEP4 cells also expressed collagen-modifying enzymes (*P4HA1*, *PLOD2*; Fig. 3b) and pro-angiogenic factor *VEGF-A*, indicating potential regulation of hepatocyte-endothelial cell interactions. Overall, the human liver demonstrates a balance between metabolic and proliferative dynamics, as also reported in mouse liver regeneration models [25].

Trajectory analysis of epithelial cells (hepatocytes and cholangiocytes) from both healthy and COVID-19 livers (Methods, Fig. 3c) suggests a differentiation path from HEP3 cells, a cell population with the highest pathway activities related to cell replication and expressing *WNT* and *NOTCH* signaling pathway genes (e.g., *TCF7L1*, *TCF7L2*, *FZD6*, *RBPJ*, *NOTCH2*; [26]) to the highly differentiated HEP2 cells, through HEP4, 1, and 5 intermediates, with HEP6 and HEP7 cell populations directly derived from HEP2. The hepatocyte population is known to be maintained both through mitosis of mature hepatocytes and differentiation from hepatic progenitor cells (HPCs) [27]. As HPCs give rise to both BECs and hepatocytes [28], and injured hepatocytes can transition into HPCs [29], we included both epithelial (hepatocyte and BEC) compartments, finding that HEP1 cells were an intermediate across hepatocytes and cholangiocytes (Fig. 3c). BEC differentiation trajectories are further discussed below.

### Hepatocyte composition and differentiation are altered in COVID-19

Contrasting healthy and COVID-19 cellular landscapes (Methods, Additional file 1: Table S5) reveals extensive remodeling of the hepatocyte compartment in COVID-19 (Fig. 3d), as well as the emergence from HEP2 cells of a COVID-19-specific HEP7 cluster, expressing acute phase proteins (Fig. 3b–d). The proportion of HEP3, a population of cells with less differentiated phenotypes, was reduced (FDR = 3.63 × 10^−54^, OR = 0.352, Binomial GLMM) whereas proportions of HEP2, HEP4, HEP5, and HEP6 cells were identified as increased (HEP2,4,5,6: FDR = 8.50 × 10^−26^, 2.37 × 10^−6^, 8.60 × 10^−6^, 2.22 × 10^−48^; OR = 1.82, 1.26, 3.04, 3.52; Binomial GLMM; respectively) or only present in COVID-19 samples in the case of HEP7 (Fig. 3c,d). Comparing the COVID-19-specific HEP7 cells to the closely related HEP6 cells, shows an inverse CEBPA/CEBPB ratio, demonstrating a metabolic vs. acute phase regulation expression program [30]. Of note, pathways such as fatty acid biosynthesis, insulin signaling, and glucose metabolism were less active in the HEP7 cluster compared to HEP2 (Additional file 2: Fig. S3c). HEP7 cells showed significant upregulation of pathways involved in immune responses and cellular stress, reflecting a shift in function from typical metabolic processing to response to infection and inflammation [31].

Notably, HEP4 hepatocytes also exhibit low *HNF4A*, *APOB*, and high *SCARB1*, *STAT3*, and *HIF1A*, a phenotype identified using bulk proteomics on severe COVID-19 patient livers, and hypothesized to be driven by the combination of hypoxia and activation of *STAT3*, leading to a reduction of the differentiated hepatocyte pathways orchestrated by downregulation of *HNF4A* [32]. The trajectory analysis reveals not only a reduction of lineages concordant to the differential cellular proportions observed, such as the increase of cells in the stressed HEP4 state, but also COVID-19-specific lineages, with high proportion of cells in the terminally differentiated HEP2 state and in the COVID-19-specific acute response HEP7 cluster (Fig. 3c, Additional file 2: Fig. S3d-e). HEP4 cells presented high expression of NF-kB, type 1 interferon signaling, and heat shock proteins, as well as an elevated autophagy activity. HEP4 cells highly expressed autophagy-related pathways and genes, such as *ATG2B*, *ATG7*, *ATG10*, *ATG5*, *ATG4C*, *ATG14*, and *MAP1LC3B*, which is an essential factor for the autophagosome formation, protein kinase AMP (*PRKAA2*), *RPTOR*, and *ULK2* which is involved in autophagy initiation (Fig. 3b).

COVID-19-specific lineage clusters, i.e., HEP2, HEP6, and HEP7, were characterized by high expression of acute phase protein genes (*SERPINA1, FGA, FGB, FGG, HP, SAA1, CRP, FTH1, C3,* Fig. 3b, Additional file 2: Fig. S2a) and by an upregulation of the unfolded protein response pathway (Additional file 2: Fig. S3a), which may predispose them to an increased response to viral infection phenotype. On the other hand, we observe an increase also in HEP2 cells which could represent a dynamic response to maintain liver function [31].

### SARS-CoV-2 RNA+ cells are enriched in hepatocyte subsets and associated with specific expression changes

We analyzed the donor- and cell type-specific distribution of SARS-CoV-2 sequencing reads to determine the presence of viral transcripts in liver cells. Specifically, we called each nucleus profile as SARS-CoV-2 RNA+ or SARS-CoV-2 RNA− by comparing the observed viral unique molecular identifier (UMI) counts to the ambient pool (a potential source of viral RNA contamination) and then tested for the enrichment of SARS-CoV-2+ nuclei in each cell type (Methods). Hepatocytes were the most enriched for SARS-CoV-2 RNA+ nuclei, particularly within the least differentiated (HEP3, 4: FDR = 1 × 10^−8^, 1 × 10^−8^; viral enrichment test; respectively) and most differentiated clusters (HEP6, HEP7: FDR = 0.040, 0.066; viral enrichment test; respectively) (Fig. 3e).

**Fig. 4:**
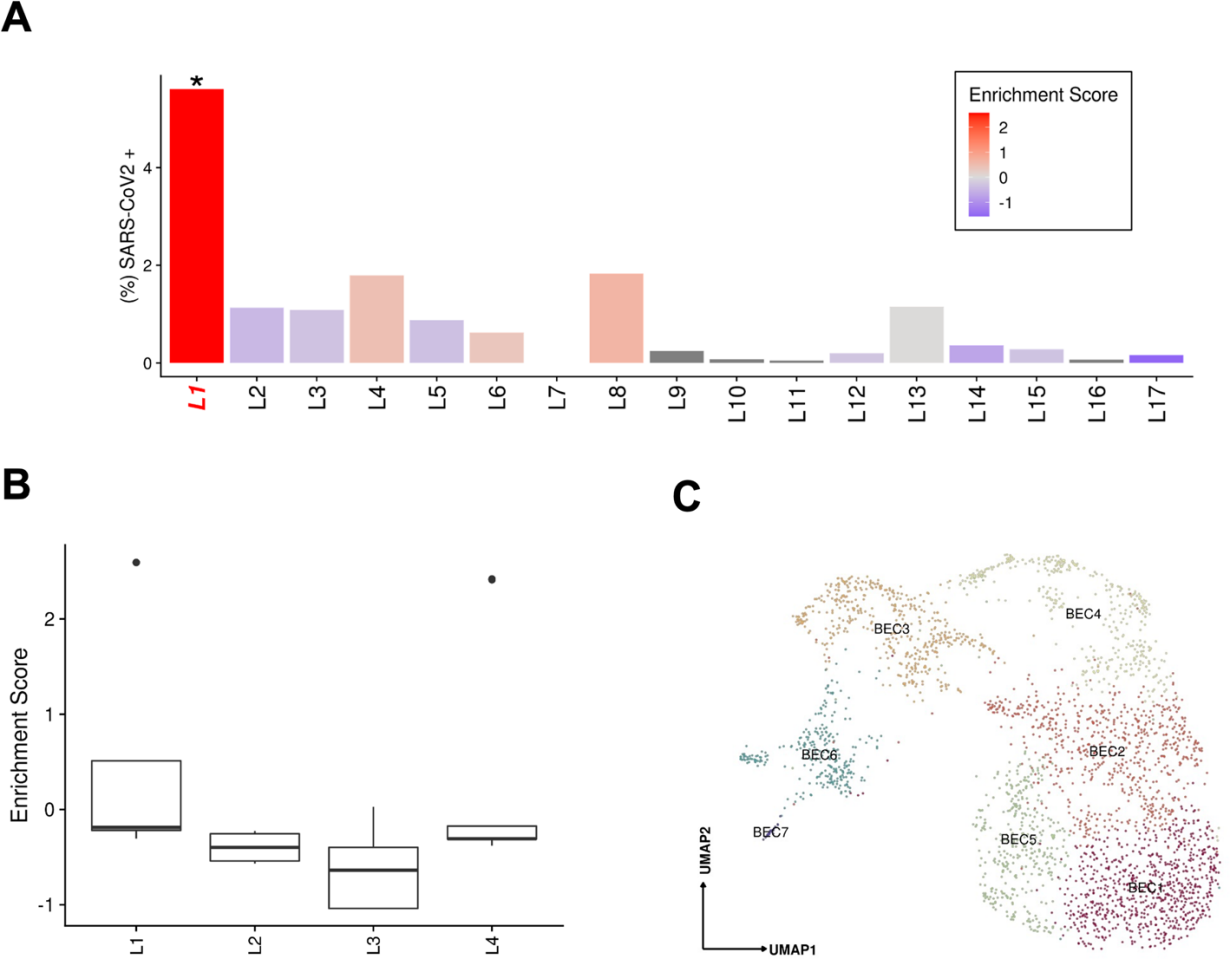
**A** Abundance of SARS-CoV-2 RNA+ nuclei in the snRNAseq data for each donor. The bars are colored by the scaled viral enrichment score estimated per donor. Only donor L1 has a significant viral enrichment score (* FDR < 0.01; viral enrichment test). **B** Distribution of the NanoString GeoMx DSP SARS-CoV-2 probe enrichment score across donors. Donor L1 has a significantly higher enrichment score (FDR = 0.037, *t*-test) compared to the rest of the donors (L2-4). **C** Uniform manifold approximation and projection (UMAP) for biliary epithelial cells (BEC1 *n* = 736; BEC2 *n* = 687; BEC3 *n* = 457; BEC4 *n* = 373; BEC5 *n* = 371; BEC6 *n* = 281; BEC7 *n* = 27)

Viral RNA levels were positively associated with the expression of multiple heat shock proteins (*HSPA1A*, *HSPA1B*, *HSPA5*, *HSPA6*, *HSPA9*, *HSPB1*, *HSPD1*), which were highest in cells in clusters HEP3, 4, 6, 7 (Fig. 3f, Fig. 4a,b), suggesting activation of unfolded protein response to cellular stress in these subsets. Heat shock protein expression was identified as localized majorly pericentrally in the matched spatial transcriptomic data (Additional file 2: Fig. S3f). In HEP4, profiles with higher viral UMIs also exhibited high *NF-kB* expression (Fig. 3f) suggesting an activation of an inflammatory response, concomitant with epithelial cell SARS-CoV-2 infection [33]. Infected cells also overlapped with high pathway activity scores (Fig. 3f, Methods) for the gene ontology (GO) term “regulation of type I interferon-mediated signaling pathway” (GO:0060338). Interferon signaling pathways were identified as enriched in a bulk RNA-seq analysis of 5 samples from SARS-CoV-2 positive livers, as characterized by PCR, when compared against 5 SARS-CoV-2 negative liver samples [4]. Pathway activity comparisons between SARS-CoV-2 positive and negative hepatocytes (Additional file 2: Fig. S3g) revealed a significantly increased activity in SARS-CoV-2-positive cells of the TNF pathway, which was also identified as enriched in infected lung epithelial cells in Delorey et al. [7]. However, despite these similarities, infected hepatocytes showed increased activity of the IL-1 pathway, which was not observed in the lung single-cell atlas, as well as increased activity of the MYC and oxidative phosphorylation pathways. MYC is involved in hepatocellular proliferation [34], and overexpression in hepatocytes leads to increased liver fibrosis [35]. Regulation of oxidative phosphorylation in hepatocytes is crucial for suppressing inflammation and proliferation [36–38]. Pathways related to bile acids were identified as significantly downregulated in infected hepatocytes. Secondary bile acids play a role in modulating inflammatory responses [39].

SARS-CoV-2 RNA+ cells and viral UMIs also varied across patients. Donor L1 cells were significantly (FDR < 0.01, viral enrichment test) enriched for SARS-CoV-2 RNA+ nuclei (ninefold higher proportion of enriched nuclei vs. average of all other donors) (Fig. 4a). Since the ability to detect viral UMIs can be affected by the total number of UMI counts and the number of genes detected (Additional file 2: Fig. S4), we also tested for enrichment in SARS-CoV-2 viral-specific probes in the extended NanoString GeoMx DSP WTA assay (Methods). Donor L1 has a significantly higher enrichment score (FDR = 0.037, *t*-test) for viral probe counts compared to the other donors (Fig. 4b). The significant enrichment in donor L1 for SARS-CoV-2 RNA in both the snRNA-seq and GeoMx DSP assays was consistent with the viral abundance estimated by quantitative RT-PCR using liver tissue from the same samples (Additional file 1: Table S6). Interestingly, the higher viral load detected by snRNA-seq, GeoMx, RT-PCR, and RNA ISH (Additional file 2: Fig. S1c) was not associated with gross abnormality of the liver tissue by conventional H&E staining. Consistent with previous reports [7, 40, 41], we found a negative, but not statistically significant correlation between the duration from symptom start to death, and the enrichment score for SARS-CoV-2 (*p*-value = 0.2852, Spearman *r* = − 0.336) (Additional file 1: Table S7).

### Pathological expansion of the cholangiocyte compartment in COVID-19

BECs (3.6% of COVID-19 patient liver nuclei, Supplement) expressed the lineage markers *CFTR*, *KRT7*, and *KRT19*, and spanned a broad spectrum, partitioning to six main subsets (Fig. 4c): two subsets of differentiated cholangiocytes (BEC1, 2), three of reactive cholangiocytes/HPCs (BEC4,5,6), and one minor subset of cholangiocyte with mesenchymal features (BEC7). BEC3 expressed highly MT genes and hepatocyte-specific markers *CPS1*, *ALB*, *HNF4A*, *C3*, *ABCB4*, which could potentially be doublets. BEC1 and 2 were closely related fully differentiated small cholangiocytes lining small caliber bile ducts [42], expressing secretin receptor *SCTR*, *BCL2*, and primary cilia genes (e.g., *BICC1*, *PKHD1*, *DCDC2*, *CTNND2*, *PKD2*, but not *CYP2E1*; Fig. 3b), while BEC1 expressed lower levels of *PDGFD*, *ZNF19*, *PAK3*, *ONECUT1*, and *CD133* compared to BEC2.

BEC4, 5, and 6 subsets each had a distinctive profile, consistent with either “reactive” cholangiocytes/hepatic progenitor cells (HPCs) or with a pro-fibrogenic “ductular reaction” in chronic liver diseases [43]. BEC4 cells comprised osteopontin-positive reactive cholangiocytes/hepatic progenitor-like cells (HPCs), expressing *SPP1*, *SOX9* [44], *LYPD6*, *CASR*, *HNF1B*, *ONECUT1*/*2*, and *GABRP*, as well as progenitor cell response genes (*ITGB6*, *FN14*/*TNFRSF12A*, *LTBR*). BEC5 were *NCAM1*^+^ immature, reactive cholangiocyte/HPCs [45], co-expressing *ITGA2*, progenitor cell markers (*SOX4*, *CK19*, *TROP2*, *CD133*), and potent pro-fibrogenic mediators (*FGF13*, *PPARD*, *PDGFC*, and *TGFB2*). BEC6 were a neuroendocrine subset of cholangiocytes [46], expressing neural markers (*TMEM132D*, *GRM7*, *HYDIN*, *NRXN3*, *LRRC4C*, *NTM*). Trajectory analysis suggests that BEC6 cells may form a potential transition state between hepatocytes and cholangiocytes (Fig. 3c), consistent with previous findings [28]. BEC7 comprised a minor subset of activated cholangiocytes co-expressing both epithelial and mesenchymal genes (*IGFBP7*, *THBS2*, *CCBE1*, *COL1A2*, *ACTA2*, *EDNRA*) and many cell–cell communication genes, especially with the endothelial compartment (*FGF*, *PDGF*, *VEGF* ligands/receptors) (Additional file 2: Fig. S5a-c), and is connected to BEC6 in the trajectory analysis (Fig. 3c).

Compared to normal liver (Fig. 3c,d), BEC4 (and BEC3s) were reduced (BEC 4,3: FDR = 2.36 × 10^−6^, 1.32 × 10^−18^; OR = 0.318, 0.162; Binomial GLMM. respectively) and BEC1, 2, 5, and 6 increased in COVID-19 liver samples (BEC1,2,5,6: FDR = 3.80 × 10^−15^, 2.22 × 10E^−5^, 7.74 × 10^−10^, 2.21 × 10^−6^; OR = 16.577, 2.736, 10.413, 11.482, Binomial GLMM), showing an extensive pathological restructuring of the cholangiocyte compartment. Spatial transcriptomics revealed that while BEC1,2 and 4 signatures mapped to portal tracts as expected (Additional file 2: Fig. S6a), HPC-like BEC6 and 7 had mixed lobular and portal distribution in COVID-19 liver, consistent with pathological “ductular reaction” expansion into the hepatic lobule [43]. We validated this observation by CK19 staining and morphometry in these livers, which revealed a presence of ductular reaction in all samples, ranging from minimal to extensive multifocal ductular proliferation extending well into the liver lobule, with up to twofold difference in CK19 + duct counts among individual livers (Donors L1-4, Additional file 2: Fig. S6b, S8).

### Kupffer cell proliferation and emergence of an erythrocyte progenitor population in COVID-19

**Fig. 5:**
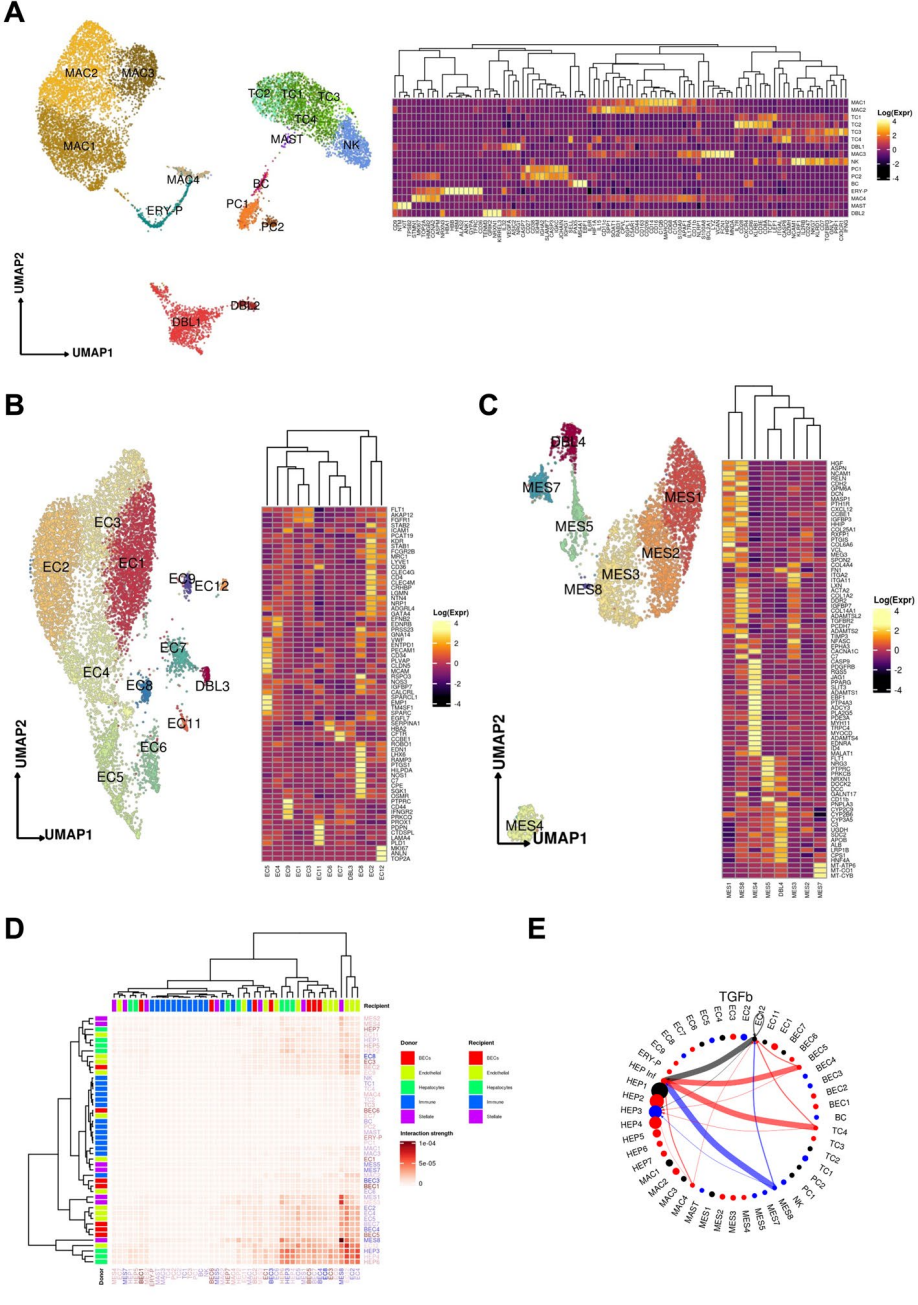
**A** Uniform manifold approximation and projection (UMAP) for the **A** immune / blood, **B** endothelial cell, and **C** mesenchymal cell compartments (**A** *Immune:* MAC1 *n* = 2798, MAC2 *n* = 2601, TC1 *n* = 1522, TC2 *n* = 388, TC3 *n* = 327, TC4 *n* = 29, DBL1 *n* = 1331, MAC3 *n* = 1038, NK *n* = 857, PC1 *n* = 397, PC2 *n* = 124, BC *n* = 124, ERY-P *n* = 359 MAC4 *n* = 222, MAST *n* = 36 DBL2 *n* = 193; **B** *Endothelial:* EC1 *n* = 2338, EC2 *n* = 2247, EC3 *n* = 1563, EC4 *n* = 1117, EC5, *n* = 795, EC6 *n* = 379, EC7 *n* = 328, EC8 *n* = 166, EC9 *n* = 116, DBL3 *n* = 91, EC11 *n* = 73, EC12 *n* = 65; **C** *Mesenchymal:* MES1 *n* = 1223, MES2 *n* = 1065, MES3 *n* = 1040, MES4 *n* = 374, MES5 *n* = 328, MES6 *n* = 312, MES7 *n* = 275, MES8 *n* = 30). Heatmaps capturing the expression of marker genes across the 3 distinct major compartments are displayed. **D** Heatmap portraying the cell–cell communications between the cell populations. The color gradient indicates the strength of interaction between any two cell groups. Recipient/Donor cell-type color is portrayed in a blue (healthy) to red (COVID-19) gradient, relevant to the cell composition fold-change differences between healthy and COVID-19 liver samples. **E** Circle plot portraying the aggregated cell–cell communication network in the TGFb pathway. This analysis includes the enriched hepatocytes in SARS-CoV-2 reads as a separate population (HEP Inf). Thicker edge lines indicate a stronger signal, while circle sizes are proportional to the number of cells in each cellular compartment. Donor edge-line and circle color are portrayed in blue (significantly increased in healthy liver samples), red (significantly increased in COVID-19 liver samples), and black (no significant difference between COVID-19 and controls in cell proportions), concordantly with the cell composition fold-change differences between healthy and COVID-19 liver samples

The immune and blood cell compartment of COVID-19 livers (15.3% of COVID-19 patient liver nuclei) spanned monocytes/macrophages/Kupffer (KCs), T cells, B cells, natural killer (NK) cells, and mast cells in diverse cellular states (Fig. 5a, Supplement).

Both the myeloid and T cell compartments were remodeled in the COVID-19 liver compared to healthy controls (Fig. 3d). Naive CD8 + T cells with high expression of *LEF1* and *TCF7* (TC1) were significantly decreased in COVID-19 liver (FDR = 1.45 × 10^−9^, OR = 0.629, Binomial GLMM), while cytotoxic effector/memory T cells (TC3), expressing *IFNγ*, *CX3CR1*, *TGFBR3*, *GNLY*, and *GZMH*, and the apoptotic naive T cell-like (TC4) population were both significantly increased in the COVID-19 liver (TC3, TC4: FDR = 1.69 × 10^−4^, 2.59 × 10^−2^; OR = 4.127,1.969, respectively, Binomial GLMM). In the myeloid compartment, there were no differences in classical Kupffer cells (KCs) (MAC1) or inflammatory KCs (MAC3) (MAC1, MAC3: FDR = 0.231, 0.154; OR = 0.925, 0.873, respectively, Binomial GLMM), but an increased proportion of MAC2 cells was observed in COVID-19 (FDR = 1.86 × 10^−2^, OR = 1.182, Binomial GLMM), an intermediate phagocytic macrophage phenotype with lower expression of *MARCO* and *CD164* but increased expression of phagocytic markers (*C5AR1*, *CPVL*, CD206). None of the macrophage subsets expressed high levels of chemokine receptors (*CCR2, CCR5, CXCR3*), indicating a deficiency of infiltrative monocyte derived macrophages, which potentially reflects a degree of immune exhaustion and/or pulmonary tropism.

The atlas also captured several proliferating cell populations that have not been previously identified in human liver single-cell studies, were nearly exclusive to COVID-19 samples, and may play important roles in regeneration. In particular, a small subset of proliferating Kupffer cells (MAC4), were significantly increased in COVID-19 livers (FDR = 7.19 × 10^−4^, OR = 3.395, Binomial GLMM) (Fig. 3d). Kupffer cells can replicate following tissue injury and were recently reported as the first cell type to enter a proliferating program in mouse liver regeneration [47], but have not been until now reported in human samples. MAC4 cells in COVID-19 liver samples recapitulate the scRNAseq signature identified in mouse liver following injury (Additional file 2: Fig. S6c-d, Methods). Moreover, erythrocyte precursors (ERY-P) were detected almost exclusively in the COVID-19 liver (FDR = 2.37 × 10^−6^, OR = 12.554, Binomial GLMM), expressing a combination of hemoglobin and glycophorin genes, proliferation genes, and additional genes not present in mature red blood cells, such as CD71/*TFRC*, which are rarely encountered outside the bone marrow in adults. These cells may be responsible for extramedullary hematopoiesis in the setting of hypoxia, modulate immune response in virus infection, and participate in hepatogenesis as shown in fetal liver [48, 49].

### Disrupted zonation and differentiation of endothelial cells in COVID-19

Cells in the endothelial compartment (11.5% of COVID-19 patient nuclei) spanned 12 subsets, including liver sinusoidal endothelial cells (LSEC) and other endothelial cell (EC) populations in an 8:1 ratio (Fig. 5b, Supplement).

Endothelial cell composition was substantially impacted in COVID-19 *vs*. healthy liver (Fig. 3d). EC1 cells, the largest endothelial subset in COVID-19 liver samples, were significantly increased in proportion compared to healthy liver (FDR = 2.76 × 10^−23^, OR = 8.63, Binomial GLMM). These cells expressed *VEGFR1*, *FGFR1*, and A-kinase Anchoring Protein 12 (*AKAP12*), but were *VEGFR2* negative. *FGFR1* is upregulated in cholestatic liver injury in mice, which provokes maladaptive fibrogenesis [50], while *AKAP12* deficiency is linked to VEGF-induced endothelial cell migration [51], regulates cell adhesion [52], and supports the integrity of the blood brain barrier during ischemic injury [53]. In the liver, *AKAP12* also modulates the activity of hepatic stellate cells (HSC) in liver injury [54]. Thus, EC1 represents a LSEC-derived profibrotic niche in response to systemic illness, either directly or indirectly from SARS-CoV-2. Conversely, EC2s, typical liver sinusoidal endothelial cells (LSEC) with high lymphatic vessel endothelial hyaluronan receptor (*LYVE1*) expression, and EC8s with features of classical vascular endothelial cells and high anti-inflammatory gene *C7* expression [55] were both significantly reduced in the COVID-19 samples (EC2, EC8: FDR = 7.10 × 10^−11^, 5.16 × 10^−29^; OR = 0.378,0.142, respectively, Binomial GLMM) (Fig. 3d). EC3s likely represented transitional states from EC2 to EC1 and were also increased in COVID-19 livers (FDR = 2.91 × 10^−19^, OR = 10.571, Binomial GLMM).

Notably, two clusters of rare cell populations were detected almost exclusively in COVID-19 livers, which may partly reflect the larger number of profiled nuclei. EC11 cells, a rare subset of *FLT1* (*VEGFR1*) negative cells (0.8% of endothelial cell nuclei; 0.09% of all profiled nuclei; FDR = 7.61 × 10^−1^, OR = 9.665, Binomial GLMM) are lymphatic endothelial cells, which are potentially captured in our COVID dataset due to the larger number of profiled nuclei. Another rare subset detected primarily in COVID-19 liver were EC12 cells (FDR = 1.76 × 10^−1^, OR = 2.864, Binomial GLMM), expressing proliferation and angiogenesis-associated genes. This subset is reminiscent of replicating endothelial cells observed in mouse lung following influenza injury [56]. Using pathway activity scores, EC12 cells clearly recapitulated the cell signature observed in influenza infected mice (Additional file 2: Fig. S6e, Methods).

### Fibrogenic activation in the mesenchymal compartment in COVID-19 patient livers

The eight subsets of mesenchymal cells (5.8% of COVID-19 nuclei) represented all major cell lineages found in the liver, including quiescent and activated hepatic stellate cells (HSCs), smooth muscle cells (SMCs), myofibroblasts (MFs), and fibrocytes (Supplement).

Mesenchymal cell proportions shifted substantially in COVID-19 liver, consistent with profibrotic HSC activation (Figs. 5c and 3d). While the proportions of quiescent HSCs (qHSCs, MES1)—the largest mesenchymal subset—were unchanged between healthy and COVID-19 livers (FDR = 0.121, OR = 0.807, Binomial GLMM), partially activated HSCs (aHSCs) (MES2) and extracellular matrix (ECM)-associated HSCs (MES3) were both significantly increased in COVID-19 livers (MES2, MES3: FDR = 1.44 × 10^−8^, 9.21 × 10^−4^; OR = 2.149, 1.508; Binomial GLMM; respectively), as were smooth muscle cells (SMCs) (MES4) (FDR = 1.66 × 10^−4^, OR = 2.181, Binomial GLMM). Conversely, both putative bone-marrow-derived fibrocytes [57] (MES5) and a minor subset of activated myofibroblasts (MES8) were decreased in proportion in COVID-19 *vs.* healthy liver (MES5, MES8: FDR = 3.28 × 10^−9^, 1.09 × 10^−2^; OR = 0.479, 0.205; Binomial GLMM; respectively). MES7 cells exhibited high expression of mitochondrial genes and low nuclear mRNA counts pointing to apoptotic cells or a technical artifact.

As expected, both MES1 (quiescent HSCs) and MES2 (activated HSCs) demonstrated translobular localization in the spatial analysis (Additional file 2: Fig. S6a), indicative of in situ activation of perisinusoidal qHSCs in response to parenchymal injury. Importantly, HSC activation was validated by immunohistochemistry for the classical HSC activation marker alpha-SMA, demonstrating a massive fibrogenic activation of HSCs across all studied livers (Additional file 2: Fig. S6b, Methods). In contrast, MES3 (ECM-associated HSCs), MES4 (SMCs), MES5 (fibrocytes), and MES8 (activated myofibroblasts) were mapped to the portal tract (Additional file 2: Fig. S6a). Surprisingly, we were not able to identify portal fibroblasts (PF) in the mesenchymal compartment based on PF-specific markers reported in the literature [58, 59]. This is consistent with evidence that collagen-producing myofibroblasts are a progeny of pericyte-like qHSCs, as suggested in fate-tracing studies in mice [60], and does not appear to support the appreciable contribution of PFs [58, 59] to the pool of fibrogenic effector cells in the human liver in the setting of subacute liver injury.

### Cellular communication networks reveal active fibrogenesis mediating altered cellular programs in COVID-19

Cell–cell communication analysis in COVID-19 donor snRNA-seq data (Methods) revealed a potential multi-cellular hub of interacting mesenchymal cells, endothelial cells, and hepatocytes (Fig. 5d, Additional file 2: Fig. S5a). The hepatocyte and endothelial compartments demonstrated signaling through the *ERBB* family of proteins, including neuregulin (*NRG*) and epidermal growth factor (*EGF*), as well as the *TGFβ* family of proteins, including the central pro-fibrogenic cytokine transforming growth factor beta (*TGF-β1*), and bone morphogenetic protein 5 and 6 (*BMP-5, -6*) (Additional file 2: Fig. S5b,c). This finding, although not reported in COVID-19-related liver pathology, is consistent with their previously reported role in liver tissue regeneration, cellular homeostasis, and extracellular matrix remodeling associated with scarring [61–66].

We identified a robust *VEGF* signaling network that predominantly emanates from the hepatocyte compartment. The high contribution by the *VEGF-A* ligand correlates with its reported upregulation under hypoxic conditions and its role in maintenance of LSEC differentiation and of liver regeneration by enhancing liver endothelial cell communication with neighboring parenchymal cells [66–69]. The *LIGHT* and *CXCL* signaling networks presented a distinguishable narrow number of cell–cell interactions with strong communication probability. Tumor necrosis factor superfamily 14 (*TNFSF14*) was the main driver of the former network with a markedly strong interaction between subclusters HEP2 and HEP5. This interaction could represent an underlying homeostatic mechanism between distinct hepatocytes responsible for regulating *TGF-β1* expression in liver fibrosis [70]. Interestingly, *TGFβ*-centric communication was observed between MES8 and HEP7 cells (a COVID-19-specific subset), suggesting stressed hepatocytes could be driving fibrogenic HSC activation. In addition, HEP7 also produces *CXCL12*, which promotes angiogenesis, inflammation, and has been shown to cause fibrogenesis in the lung [71] (Additional file 2: Fig. S5b, c).

Finally, we evaluated the role of the infected hepatocytes (HEP Inf) in the cellular communications network. A cell–cell communication analysis was performed following the selection of the SARS-CoV-2 viral read-enriched hepatocytes as a distinct population (Methods). The analysis revealed active autocrine signaling among the HEP Inf cells and strong interactions with other hepatocyte, mesenchymal, cholangiocyte, and endothelial populations (Additional file 2: Fig. S7a). HEP Inf cells were identified as a dominant signaling hub for a *TGFβ* communications network comprising endothelial, cholangiocyte, and immune cells (Fig. 5e, Additional file 2: Fig. S7b), consistent with previous reports related to chronic immune reaction [72], and *TGF-β* implication in inflammation and liver fibrosis. Likewise, potential susceptibility to live fibrosis was underlined by additional cell interactions such as through the complement factor C5 (*HC*) network, previously discussed for its implication in liver fibrosis in mouse models [73, 74] (Additional file 2: Fig. S7c-d). This analysis also revealed a dense cell–cell interaction network between activated immune cell populations and HEP Inf, through the *SEMA4A* pathway, which can regulate immune cell activation and differentiation [75].

Overall, the identified cell–cell communication pathways support a diverse source of fibrogenic activation, involving hepatocytes, cholangiocytes, endothelial and immune cells, in contrast to an immune cell dominated framework seen in many chronic liver diseases.

### Histopathology validation of an extensive pro-fibrotic cellular phenotype of COVID-19 livers

To validate the insights from our atlas, we performed a liver histopathology survey in the liver tissue available to us from the four cases (Donors L1-4, Methods), where snRNA-seq and GeoMx assays were performed. Surprisingly, a common striking pathology feature of all four COVID-19 livers was the stellate cell activation and sinusoidal fibrosis, ranging from moderate in L1 to massive in L4. Upon further review of the medical records, none of the four donors had a history of chronic liver disease or clinical evidence of ongoing liver injury in the 72 h prior to death. Three out of four patients also demonstrated moderate to extensive ductular reaction/cholangiocyte proliferation (Additional file 2: Fig. S6b, Additional file 1: Table S8). This is consistent with the increased proportion of activated/transdifferentiated mesenchymal and cholangiocytic cell subsets identified in our snRNA-seq. Although pro-fibrogenic and HSC activation pathways were observed in the cell–cell communication analysis, they cannot completely explain the great extent of HSC activation observed in the histopathological analysis. Thus, extrahepatic, systemic signals may additionally contribute to the activation of HSCs and fibrosis in the liver of severe COVID-19. Since severe COVID-19 has features of an atypical viral sepsis-like condition that goes on for an extended period of time [76], our findings therefore share features of the low-grade inflammation, stellate cell activation, ductular reaction, and hepatic fibrosis observed in experimental sepsis in mice [77].

## Discussion

We have generated a cellular and spatial atlas of the COVID-19 liver by integrating snRNA-seq and spatial transcriptomics on autopsy samples obtained from patients who died from COVID-19. We acquired > 80,000 high-quality single-nucleus profiles with > 50% hepatocyte representation, providing us with a rich, granular dataset, even for rare cell subsets.

We observed extensive pathological restructuring of the cellular and expression landscape in COVID-19 livers, suggesting hepatocellular injury, ductular reaction, neo-vascular expansion, and fibrogenesis. Based on viral RNA reads, we identified human hepatocytes infected by SARS-CoV-2 and characterized their expression profiles, while also capturing indirect and systemic effects of COVID-19 on hepatocyte populations. The highest number of SAR-CoV-2 viral RNA UMIs was found in hepatocytes, while a previously proposed cholangiocyte-tropism [78] in the liver was not seen. Viral RNA UMI-enriched hepatocytes exhibited high expression of acute phase and pro-inflammatory proteins, with increased heat shock protein gene expression, likely a response to unfolded proteins, secondary to viral replication; and *NF-kB* expression, consistent with the [79] available literature for other epithelial cell types [79]. Our results also recapitulated the observation of high Interferon signaling pathway activity, as were suggested in a bulk RNA-seq analysis of 5 samples from SARS-CoV-2 positive livers compared against 5 SARS-CoV-2 negative liver samples [4]. Elevated proportions of SARS-CoV-2 + hepatocyte populations were also noted, with high abundances of cells in the terminally differentiated states as well as the emergence of a SARS-CoV-2 + -specific cluster (HEP7) with a shift in function from metabolic processing to response to infection and inflammation. Terminally differentiated hepatocytes demonstrated both high metabolic activity and an increased inflammatory response, characterized by high expression of acute phase proteins, which may enable the increased response to viral infection pathway activity observed in SARS-CoV-2 + cells of these populations [80]. Additionally, the increased abundance of these cells in COVID-19 patients, along with their elevated typical metabolic processes, may reflect a compensatory mechanism to maintain liver function and address the increased metabolic demands and detoxification needs during disease progression [31]. On the other hand, viral-infected hepatocytes not included in the COVID-19-specific lineage maintained a highly stressed state and elevated autophagy activity. These populations presented high expression and activity of autophagy-related pathways and genes, which could represent an effort of the cells to induce clearance of viruses through viral component encapsulation in autophagosomes, and lysosomal degradation. However, coronaviruses have been shown to turn autophagy into a double-edged sword, and through its modulation, they can prevent degradation, further enhancing their replication and persistence within host cells [81, 82].

Meanwhile, profibrogenic/reactive cholangiocytes were identified as characteristic populations expanding in the COVID-19 liver, representing a pathological “ductular reaction”—an extensive remodeling and scarring of biliary compartment, secondary to local as well as systemic liver injury [43]. This striking observation was validated by connective tissue stains and conventional immunohistochemistry and morphometry for CK19 + ducts, clearly showing various degrees of ductular reaction and associated a-SMA + myofibroblast activation, consistent with emerging reports of COVID-19-induced sclerosing cholangitis (fibrotic disease of bile ducts) [83], which in most severe cases may require liver transplantation [84]. Our high-resolution observations and comprehensive molecular characterization of cell–cell interactions in subacute COVID-19 cholangiopathy may provide a unique opportunity to elucidate relevant drivers of other chronic cholangiopathies of enigmatic nature and currently without effective therapy, such as primary sclerosing cholangitis (PSC), which is challenging to study due to slow progression and scarce opportunities to analyze the liver tissue in early disease stages. In this respect, we have identified several mediators (TGFbeta, PDGF) that were well studied in cholangiopathies such as PSC as well as those that are much less explored, such as VEGF. Since several VEGF inhibitors were developed, rapid functional and therapeutic validation of this new target is feasible and is an area of our future studies.

We also found extensive changes in the composition and expression programs of non-parenchymal cells across the liver lobule and portal triad in COVID-19. Endothelial cell population proportions are significantly altered in COVID-19 livers, with the emergence of a large population of *FGFR1* and *AKAP12-*positive cells that may contribute to angiogenesis and promote fibrosis [85, 86]. In the immune compartment of the COVID-19 liver, we observed KC proliferation and erythrocyte progenitors for the first time in a human single-cell study. We also observed activation of mesenchymal stellate cell/myofibroblast cells both in the liver lobule and portal areas, which were validated by immunohistochemistry staining, and an expansion of smooth muscle cell population in the COVID-19 liver samples. This pattern of fibrosis cannot be explained by underlying chronic liver disease and is likely caused by a combination of localized and systemic, sepsis-like effects of severe COVID-19 [77]. These cellular and expression changes induced by COVID-19, despite an absence of significant tissue injury, point to subclinical yet profound effects of COVID-19 on the human liver, and may carry long-term health implications for those who recover from acute infection.

Our study captured the complexity of liver biology at high resolution, providing new insights into cellular plasticity and regeneration in the liver. Based on their RNA expression profiles, a significant proportion of the hepatocytes do not appear to contribute directly to liver function by conventional definitions, while reflecting other processes such as cellular differentiation, growth, and wound healing. Compared to previous single-cell studies, we did not observe a strict zonated distribution of hepatocyte clusters; our spatial data suggest several hepatocyte subtypes may intercalate in a mosaic pattern, which may have biological advantage in liver injury and regeneration. Whether this can be generalized in healthy liver as well or an observation only specific to COVID-19 needs to be further studied. Similarly, in the BEC compartment, we characterized rarely identified cells, such as neuroendocrine cholangiocytes, and a bidirectional trajectory axis between cholangiocytes and hepatocytes with specific cell transition states between these cell types, not previously reported in human samples. These specific changes in BEC and MES compartment strongly suggest activation of fibrogenic response, and appear to be consistent with recently reported sclerosing cholangitis-like sequelae of COVID-19. Other hematopoietic lineage cells were found to be in a proliferative state, including erythrocyte progenitors and plasmablasts. The former are not commonly encountered outside the bone marrow in adults, while the latter further support the recent observations made by Dominguez Conde et al. [87] showing the presence of this population along with ITGA8-positive plasma cells in the human liver.

In this study, single-nucleus sequencing was an advantageous modality, supporting highly standardized and streamlined sample collection in very demanding circumstances as opposed to single-cell sequencing, where fresh samples are required to be readily processed. Furthermore, it supported the generation of a large dataset with > 80 K nuclei passing QC, with a representation of parenchymal and non-parenchymal cell populations resembling more liver physiology as opposed to single-cell sequencing, where lymphocyte populations are often largely overrepresented [16, 88]. Our study was limited by including a relatively small number of patients (*n* = 17) with a severe post-acute COVID-19 phenotype, not enabling us to directly assess moderate and less severe or acute manifestations of the disease. We plan on building upon the generated information with studies comprising a larger and more heterogeneous population.

Furthermore, it is important to note that all analyses were performed in a limited section of the patient livers due to the organ’s extensive size and the limitations of current single-cell and spatial tissue profiling technologies. However, the amount of tissue sampled in our study via 13G core biopsy is comparable to or greater than a standard 1.5-mm liver needle biopsy used in most studies of human liver pathophysiology. Future studies expanding the current study design could be very useful to identify further phenotypic and mechanistic differences, including liver tissue samples not only from healthy controls and COVID-19 patients but also from long COVID patients who succumbed to causes unrelated to COVID-19, as well as from patients with non-COVID-19-related pneumonia/ARDS. As all samples were analyzed early in the pandemic, they cannot inform impact from vaccination, and reflect only the very early lineages of the virus. Nevertheless, this extensive dataset offered unique insights on the sub-clinical COVID-19 liver phenotype and biology, while its very high granularity and complementary methods enable it to become the foundation of future meta-analyses and could complement basic, clinical, and translational research efforts. Importantly, this investigation focused on the generation and in-depth analysis of the single-cell and spatial tissue profiling data from the collected tissue samples, on the comparisons between healthy controls and COVID-19 patients, as well as on the transcriptional phenotyping of SARS-CoV-2-positive cells. However, these observations have not been followed by downstream experimental investigations, which is a limitation. Nevertheless, single-cell and spatial-omic profiling studies of patient tissues have extended our understanding of the cell- and tissue-specific effects of COVID-19, by providing a highly granular characterization of the cellular populations, tissue architecture, and interactions in a hypothesis-free manner, offering novel insights and fueling reverse translation [7, 89–91]. We hope that the generated data from this atlas of the post-acute severe COVID-19 liver could enable follow-up mechanistic studies but also the generation of targeted diagnostic and intervention strategies.

## Conclusions

Our study revealed SARS-CoV-2 RNA-positive hepatocytes with an expression phenotype similar to infected lung epithelial cells, and central in a pro-fibrotic TGFβ signaling cell–cell communication network. We performed integrated analysis and comparisons with healthy control that revealed extensive changes in the cellular composition and expression states in COVID-19 liver. These findings serve as the basis for understanding the underpinning of hepatocellular injury, ductular reaction, pathologic vascular expansion, and fibrogenesis characteristic of COVID-19 cholangiopathy. Our study identified several suspected (TGFb, PDGF) and novel (VEGF) potential molecular drivers of COVID-19 cholangiopathy, with potentially far-reaching translational and pharmacological implications for biliary diseases. Moreover, we made a novel observation of Kupffer cell proliferation and the presence of erythrocyte progenitors in a human liver cell atlas. In COVID-19, the endothelial cell composition was dramatically impacted despite the lack of clinical acute liver injury phenotype. This was accompanied by extensive alterations and fibrogenic activation of reactive cholangiocytes and mesenchymal cells.

This investigation focused on the generation and in-depth analysis of the single-cell and spatial tissue profiling data from the collected tissue samples, on the comparisons between healthy controls and COVID-19 patients, as well as on the transcriptional phenotyping of SARS-CoV-2-positive cells. However, these observations have not been followed by downstream experimental investigations, which is a limitation. Nevertheless, single-cell and spatial-omic profiling studies of patient tissues have extended our understanding of the cell- and tissue-specific effects of COVID-19, by providing a highly granular characterization of the cellular populations, tissue architecture, and interactions in a hypothesis-free manner, offering novel insights and fueling reverse translation [1–4]. We hope that the generated data from this atlas of the post-acute severe COVID-19 liver could enable follow-up mechanistic studies but also the generation of targeted diagnostic and intervention strategies.

## Methods

### Patient cohorts

An autopsy cohort of 17 COVID-19 patients (6 males, 11 females, ages from 30–35 to > 89) was collected from 4 medical centers from the Northeastern United States during the first wave of the pandemic (Table 1). For all patients, consent was acquired by their healthcare proxy or next of kin prior to their inclusion to the study. Exclusion criteria included high post mortem interval (> 24 h) and HIV infection. All samples were obtained post mortem using either ultrasound-guided needle biopsy or surgical dissection. All sample collection procedures were reviewed by the IRB of the relevant hospital. The related protocols were as follows: Beth Israel Deaconess Medical Center (IRB 2020P000406, 2020P000418), Brigham and Women’s Hospital and Massachusetts General Hospital (2020P000804, 2020P000849, 2015P002215), New York Presbyterian Hospital/Columbia University Medical Center (IRB-AAAT0785, IRB-AAAB2667, IRB-AAAS7370). All patients had confirmed COVID-19 by PCR testing. Consent for autopsy and research was obtained from the healthcare proxy or the next of kin. Massachusetts Institute of Technology (MIT) IRB protocols 1,603,505,962 and 1,612,793,224, and/or the not-involving-human-subjects research protocol ORSP-3635, cover all secondary analyses performed at the Broad Institute of MIT and Harvard. No subject recruitment or ascertainment was performed as part of the Broad protocol. Donor identities and accompanying information were encoded at the relevant hospital site prior to shipping to or sharing with the Broad Institute for sample processing or data analysis. We also included snRNA-seq data from snap-frozen biopsies from 4 healthy neurologically deceased donor livers suitable for transplantation (G.B., S.A.M), age 40–49 (F), age 40–49 (M), age 40–49 (F), and age 20–29 (F) (Table 1).

### Sample acquisition

#### Beth Israel Deaconess Medical Center (BIDMC)

Sample collection for BIDMC samples was performed by an interventional radiologist via a 13G coaxial guide with a 14G core biopsy and 20-mm sample length under ultrasound guidance. All biopsies were conducted within 3 h of confirmed asystole on a gurney in the hospital morgue. All personnel were wearing standard personal protective equipment prior to removing the body from the bag. Multiple biopsies were acquired by tilting the coaxial needle a few degrees in different directions. Core biopsies were separated in two groups: one for formalin fixing and the other flash-frozen in liquid nitrogen and stored at − 80 °C until use.

#### Brigham and Women’s Hospital (BWH)

Sample collection for BWH was performed in a negative pressure isolation room with personnel wearing personal protective equipment (powered air-purifying or N95 respirators). Abdominal organs were harvested en bloc and the liver was then subsequently dissected, weighted, and photographed. Liver samples were collected from the organ and placed in 25 mL of RPMI-1640 media with 25 mM HEPES and L-glutamine (Thermo Fisher Scientific) + 10% heat inactivated FBS (Thermo Fisher Scientific) in 50-mL falcon tubes (VWR International Ltd). Tissue samples were transported to Broad in a cooler.

#### Massachusetts General Hospital (MGH)

Sample collection for MGH was performed in a negative pressure isolation room from personnel wearing personal protective equipment (N95 or powered air-purifying respirators). As in BWH, organs were removed en bloc, dissected, photographed, and weighed. Liver samples were placed in collection tubes and subsequently in a cooler for transport to the Broad Institute.

#### New York Presbyterian Hospital

Sample collection was performed as in [8]. Tissue samples were collected during rapid autopsy within hours from time of death. Tissue samples of ~ 1 cm^3^ were embedded in Tissue-Tek optimal cutting temperature (OCT) compound (Sakura Finetek USA Inc) and stored at − 80 °C.

### Tissue processing and single-nuclei encapsulation

All samples from all hospitals were snap frozen for the snRNA-seq studies. All sample handling steps were performed on ice. TST and ST buffers were prepared fresh as previously described [92, 93]. A 2 × stock of salt-Tris solution (ST buffer) containing 292 mM NaCl (Thermo Fisher Scientific), 20 mM Tris–HCl pH 7.5 (Thermo Fisher Scientific), 2 mM CaCl_2_ (VWR International Ltd), and 42 mM MgCl_2_ (Sigma Aldrich) in ultrapure water was made and used to prepare 1xST and TST. TST was then prepared with 1 mL of 2 × ST buffer, 6 µL of 10% Tween-20 (Sigma Aldrich), 10 µL of 2% BSA (New England Biolabs), and 984 µL of nuclease-free water 1xST buffer was prepared by diluting 2 × ST with ultrapure water (Thermo Fisher Scientific) in a ratio of 1:1. One milliliter of PBS-0.02% BSA was also prepared with 990 µL UltraPure 1 × PBS ph 7.4 (Thermo Fisher Scientific) and 10 µL 2% BSA (New England Biolabs) for sample resuspension and dilution prior to 10 × Genomics chip loading. Single frozen biopsy pieces were kept on dry ice until immediately prior to dissociation. With clean forceps, a single frozen biopsy was placed into a gentleMACS C tube on ice (Miltenyi Biotec) containing 2 mL of TST buffer. gentleMACS C tubes were then placed on the gentleMACS Dissociator (Miltenyi Biotec), and tissue was homogenized by running the program “m_heart_02” × 2 until tissue was fully dissociated. A 40-µm filter (CellTreat) was placed on a 50-mL falcon tube (Corning). Homogenized tissue was then transferred to the 40-µm filter and washed with 3 mL of 1xST buffer. Flow-through was transferred to a 15-mL falcon tube (Corning). Samples were then centrifuged at 500* g* for 5 min at 4 °C with brake set to “low”. Sample supernatant was removed, and the pellet was resuspended in 100–200 µl PBS-0.02% BSA. Nuclei were counted and immediately loaded on the 10 × Chromium controller (10 × Genomics) for single-nucleus partitioning into droplets.

### Single nuclear RNA sequencing

For each sample, 8000–16,500 nuclei were loaded in one channel of a Chromium Chip (10 × Genomics). 3’ v3.1 chemistry was used to process all other tissues. cDNA and gene expression libraries were generated according to the manufacturer’s instructions (10 × Genomics). cDNA and gene expression library fragment sizes were assessed with a DNA High Sensitivity Bioanalyzer Chip (Agilent). cDNA and gene expression libraries were quantified using the Qubit dsDNA High Sensitivity assay kit (Thermo Fisher Scientific). Gene expression libraries were multiplexed and sequenced on an Illumina sequencer.

### SnRNA-seq expression quantification and correction for ambient RNA

The raw sequencing reads were demultiplexed using Cell Ranger mkfastq (10 × Genomics). We trimmed the reads from the BIDMC liver samples for polyA tails and the template switching oligo 5′- AAGCAGTGGTATCAACGCAGAGTACATrGrGrG -3′ with cutadapt v.2.7 [94]. The reads were aligned to generate the count matrix using Cell Ranger count (10 × Genomics) on Terra with the cellranger_workflow in Cumulus [95]. The reads were aligned to a custom-built Human GRCh38 and SARS-CoV-2 (“GRCh38_premrna_and_SARSCoV2”) RNA reference. The GRCh38 pre-mrna reference captures reads mapping to both exons and introns [92]. The SARS-CoV-2 viral sequence (FASTA file) and accompanying gene annotation and structure (GTF file) are as previously described [96]. The GTF file was edited to include only CDS regions, with added regions for the 5′ UTR (“SARSCoV2_5prime”), 3′ UTR (“SARSCoV2_3prime”), and anywhere within the Negative Strand (“SARSCoV2_NegStrand”) of SARS-CoV-2. Trailing A’s at the 3′ end of the virus were excluded from the SARSCoV2 fasta file [7]. CellBender remove-background [97] was run to remove ambient RNA and other technical artifacts from the count matrices. The workflow is available publicly as cellbender/remove-background (snapshot 11) and documented on the CellBender GitHub repository as v0.2.0: https://github.com/broadinstitute/CellBender.

### Filtering of low-quality cells and sample integration

We filtered out nuclei with fewer than 400 UMIs, 200 genes, or greater than 20% of UMIs mapped to mitochondrial genes. Furthermore, we discarded samples with less than 100 nuclei. We retained all nuclei that pass the quality metrics described above. Subsequently, snRNA-seq data from individual samples were combined into a single expression matrix and analyzed using Seurat v.3.2.3 [98–100]. The UMI counts for each nuclei were divided by the total counts for that nuclei, and multiplied by a scale factor of 10,000. Then, values are log-transformed using log1p resulting in log(1 + 10,000*UMIs/Total UMIs) for each nucleus.

Subsequently, highly variable genes were identified using Seurat’s FindVariableFeatures function. Then, data dimensionality was reduced to the top 15 principal components by PCA using the top 2000 highly variable genes. The lower dimensional embedding was then corrected for technical noise using each sample as a separate batch with Harmony [101]. Neighbors were computed using the Harmony-corrected embedding. The UMAP and Leiden clusters were computed using the resulting nearest neighbor graph.

### Doublet detection

We used a two-step procedure to identify doublets. First, we identified doublets in each sample with the re-implementation of the Scrublet [102] algorithm in Pegasus [7, 95]. Second, we integrated and clustered all samples and identified clusters significantly enriched for doublets. All nuclei in the enriched clusters were flagged as potential doublets.

In brief, we integrated the nuclei that passed the quality control, normalized each nuclei to feature counts per 100 K counts (FP100K) and log transformed the expression values (log(FP100k + 1)), selected highly variable genes, computed the first 30 principal components (PCs), corrected the PCs for batch effects using Harmony, and clustered the cells using the Harmony corrected embedding with the Leiden algorithm. Then, we tested if each cluster is significantly enriched for doublets using Fisher extract test controlling at a false discovery rate of 5%. Among the significantly enriched clusters, we selected those with more than 60% of nuclei identified as potential doublets and marked all nuclei in these clusters as doublets.

### Clustering

We first derived compartments, high-level clusters, encompassing major cell types. Then, we performed iterative clustering to identify cell types. We used the first 15 PCs corrected by Harmony to compute the nearest neighbor graph. Then we identified the compartments using the Leiden algorithm implemented in the FindClusters function in Seurat. For each compartment, we subsetted the nuclei, selected highly variable genes, computed the first 15 PCs, corrected the PCs for batch effects using Harmony, computed the nearest neighbor graph with the Harmony embedding, and clustered the nuclei using the FindClusters function in Seurat.

### Batch effect correction

Building on approaches that use residuals from a negative binomial generalized linear model (NB-GLM) to normalize single-cell data [103–105], we fitted a NB-GLM using an efficient implementation of a Gamma-Poisson GLM [19, 106] with batch as the covariates. We then used the deviance residuals from this model as the expression adjusted for batch effects. For downstream analysis that required counts, we also generated counts corrected for batch by expanding and scaling the model described by [18] using a scalable implementation of a Gamma-Poisson GLM [19].

### Pathway activity score calculation

A pathway score summarizes the expression of a set of functionally related genes [107]. A Gene Ontology [108] set of 989 GO Biological Process terms was used to create a curated selection of pathways capturing liver parenchymal and non-parenchymal cellular functions and pathways (Additional file 1: Table S9). Building on the methodology described in [107, 109], we used a rank-based approach to define the pathway scores, where the pathway score is the sum of the adjusted ranks of the genes in the pathway annotation scaled by the square root of the number of genes in the pathway. First, the ranks based on the UMI counts are calculated per gene for each nucleus solving ties by selecting the minimum. Then, we scale and center the ranks across each nucleus. In order to account for the effect of rank sparsity for each gene we split the scaled and centered ranks by their sign (positive or negative) and regress out with a linear model the effect of the number of genes detected and the log of the total number of UMIs. Finally, we use the removeBatchEffect function from limma [110] to adjust the pathway scores for batch effects. The same approach was used to estimate a score for the curated signatures described by Sánchez-Taltavull et al. (proliferating Kupffer cells) [47], and by Niethamer et al. (influenza-injury signature) [56].

### Differential expression analysis at cluster level

Differential expression analysis was carried out using limma-trend [111, 112] to detect cluster gene markers. First, genes expressed in at least 5% of the nuclei of at least one cluster were selected and then UMI counts were normalized using the TMM normalization [113] implemented in edgeR v.3.28.1 [114]. Then, a linear model “ ~ Cluster + Batch” was fitted and modeled the mean–variance relationship with the limma-trend method [111] and a robust empirical Bayes procedure [115]. We used contrasts to compare the mean of a given cluster with all others; a gene is considered a cluster marker if the contrast is significant at an FDR < 0.05 and the cluster coefficient is higher than at least 75% of all other clusters. We performed comparisons at two levels: across all compartments (comparing all clusters identified) and within compartments (comparing clusters only from the same high-level cluster). We used limma to fit the same model “ ~ Cluster + Batch” on the pathway scores but without the mean–variance trend since the pathway scores are approximately normally distributed. The criteria to select pathway markers were identical to the cluster markers.

### Healthy reference comparison and differential gene expression

We combined the COVID-19 liver nuclei passing QC and were not marked as doublets with the control liver snRNA-seq dataset into a single expression matrix. Similarly to the COVID-19 snRNA-seq analysis, we normalized each nucleus to TP100K and log transformed the expression values (log(TP100k + 1)), selected highly variable genes, computed the first 30 principal components (PCs), corrected the PCs for batch (we considered each sample as a separate batch) using Harmony, and clustered the cells using the Harmony corrected embedding with the Leiden algorithm. We identified 5 high-level compartments in the combined data set. These high-level clusters matched the compartments identified in the COVID-19 liver data. For each high-level cluster, the first 15 PCs were corrected for batch effects using Harmony and the nearest neighbor graph was calculated using the Harmony embedding. The nearest neighbor graphs were used to assign each nucleus from the healthy reference to the relevant cluster.

Differential expression analysis was carried out using limma and mean–variance modeling at the observational level (voom) [111] after summing nuclei per cluster per sample [116], and the linear model “ ~ Disease + SVs”, where SVs are surrogate variables estimated with iterative adjusted surrogate variable analysis (IA-SVA) [117]. The model was fit to estimate the differences between COVID-19 and healthy livers for each cluster. All clusters with at least 3 samples per group with > 5 nuclei per sample were included in the analysis.

### Determination of significant changes in cell type proportions

A binomial generalized linear mixed model (GLMM) was utilized to study the differences in cell type abundances between COVID-19 and control livers. Lme4 version 1.1–27.1 was utilized to fit the model ~ Cluster*Condition + (1|Sample), and emmeans version 1.6.2–1 to compare the odds ratios of COVID-19 vs Control for each cluster (Additional file 1: Table S5).

### Detection of cells with SARS-CoV-2 content above ambient levels

We adapted methods [97, 118, 119] previously described in [7] to designate a single nucleus as SARS-CoV-2 RNA + or SARS-CoV-2 RNA − . A permutation test was utilized to determine the probability that the nucleus contained a higher SARS-Cov-2 UMI content than expected by ambient contamination, while taking into account the fractional abundance of SARS-Cov-2 aligning UMIs, the abundance of SARS-Cov-2 aligning UMIs in the ambient pool, and the estimated ambient contamination of the single nucleus.

The fractional abundance of SARS-Cov-2 aligning UMIs per nucleus was defined as the number of UMIs assigned to all viral genomic features divided by the total number of UMIs aligning to either the SARS-Cov-2 or GRCh38 reference. The abundance of SARS-Cov-2 UMIs in the ambient pool was defined as the sum of all SARS-Cov-2 UMIs in the pre-CellBender output within discarded nuclei flagged as “empty” or “low quality.” Hence, the ambient fractional abundance was determined for each sample independently. The discarded nuclei were resampled to generate the null distribution of the SARS-CoV-2 fractional abundance, which was utilized to extract empirical *p*-values for the observed fractional abundance of each nucleus. The empirical *p*-values were adjusted for multiple comparisons using false discovery rate. Nuclei with at least 2 SARS-Cov-2 UMIs and an FDR < 0.05 were assigned as “SARS-CoV-2 RNA + ”; “SARS-Cov-2 Ambient” if having SARS-CoV-2 UMIs but were not significantly higher than the ambient pool; and “SARS-CoV-2 RNA − ” if no SARS-Cov-2 UMIs were detected.

### Differential expression analysis between SARS-Cov-2 RNA + and SARS-Cov-2 RNA − nuclei

In order to test the genes and pathways associated with the presence of SARS-Cov-2 RNA, we used the following approach to account for the biases due to differences in number of nuclei, quality, and sample-to-sample variability. First, we only considered cell types with at least 10 SARS-Cov-2 RNA + nuclei (above ambient levels) and within a given cell type we only considered samples with at least 2 SARS-Cov-2 RNA + nuclei. Then we subsampled the SARS-Cov-2 RNA − nuclei to match the complexity distributions. The nuclei were partitioned into 5 bins based on complexity, log10(Number of genes/nuclei), and the SARS-Cov-2 RNA − nuclei were subsampled to match the distribution of the SARS-Cov-2 RNA + nuclei [9]. We resampled the pool of SARS-Cov-2 RNA − nuclei to generate the null distribution for the mean expression and the pathway scores in order to estimate an empirical *p*-value for the mean expression in the SAR-Cov-2 RNA + nuclei. Mean expression was calculated by normalizing the UMI counts using the trimmed mean of M-values (TMM) normalization [113] and adjusted for batch effects using limma’s removeBatchEffect function. Pathway scores were estimated for the selected nuclei and then adjusted for batch effects using limma’s removeBatchEffect function. *P*-values were adjusted for multiple comparisons using FDR.

### Viral enrichment analysis

A viral enrichment score per cluster was calculated as previously [7, 120]. The enrichment score for a given cluster C is defined as follows: EnrichmentI = log( ( Observed( Vcells in C) + **ε**) / ( Expected( Vcells in C) + **ε**)) = log( ( Vcells in C) + **ε**) / ( ( Vcells in total * X_c) + **ε**) where Vcells are the SARS-Cov-2 RNA + nuclei, X_c is the proportion of the total number of nuclei in cluster C out of the total number of nuclei in its corresponding compartment, and **ε** = 0.0001. We only considered samples with at least 5 SARS-Cov-2 RNA + nuclei. We derived the null distribution of each enrichment score by permuting the data and assigning the same number of SARS-Cov-2 RNA + labels to nuclei, such that the overall proportion of SARS-Cov-2 RNA + nuclei was fixed, computing the cluster enrichment score and estimating the empirical *p*-value as the fraction of the permutations that showed a similar or higher enrichment score compared to the observed enrichment score. Then, we adjusted the empirical *p*-values for multiple comparisons using FDR.

### Trajectory interference and cell–cell communication analysis

Single-cell pseudotime trajectory was constructed using Slingshot (version 2.0.0) based on the Harmony embedding matrix. The embedding matrix was re-computed for the Hepatocyte and Biliary Epithelial cells, excluding the BEC3 doublet cluster, while the first 20 dimensions were utilized for the subsequent analysis. Lineages were determined and mapped to the UMAP embedding matrix using the relevant Slingshot protocol [121]. Cell–cell communication among the distinct cell populations was defined using the CellChat R package [122]. The average gene expression per cell group was calculated by applying a threshold of 20% and using the batch-corrected counts. Significant ligand-receptor interactions and pathways were retained by applying a 0.05 *P* value cutoff. A similar approach was followed for the cell–cell communication analysis among the distinct cell populations and the enriched hepatocytes in SARS-CoV-2 reads (HEP Inf). Specifically, significantly enriched cells for Sars-CoV-2 viral reads were marked as HEP Inf and subsequently removed from the HEP1-HEP7 cell populations prior to repeating the analysis.

### Digital spatial profiling

Liver tissue sections of 5 µm were prepared from formalin-fixed paraffin-embedded blocks. Tissue integrity was confirmed on slides stained with hematoxylin and eosin (H&E). Slides were stored in vacuum at 4 °C to preserve RNA integrity. To prepare the slides for digital spatial profiling (DSP), slides were stained against Pan-Cytokeratin, CD68, CD45, and DNA. A Whole Transcriptome Atlas (WTA) probe library (NanoString) was applied on each slide according to the manufacturer instructions. Four categories of area of interest (ROI) for transcriptome profiling were manually selected under a fluorescence-microscope: portal area, and lobular zones 1–3.

Specifically, autopsy FFPE tissues from COVID-19-infected patients were processed following the GeoMx DSP slide prep user manual (MAN-10087–04). Autopsy slides were baked in an oven at 65 °C for an hour and then they were processed on a Leica Bond RX automation platform with a protocol including three major steps: (1) slide baking, (2) antigen Retrieval 20 min at 100 °C, (3) 1.0 µg/ml Proteinase K treatment for 15 min. Subsequently, the slide was incubated with the RNA probe mix (WTA and COVID-19 spike-in panel, Additional file 1: Table S2). After overnight incubation, slides were washed with buffer and stained with CD68-594 (Novus Bio, NBP2-34736AF647), CD45-647 (Novus Bio, NBP2-34527AF647), PanCK-488 (eBioscience, 53–9003-82), and Syto83 (Thermo Fisher, S11364) for 1 h, and loaded on the NanoString GeoMx DSP to scan 20X fluorescent images. Regions of interest (ROIs) were placed by an expert panel comprising hepatologists, pathologists, and technology specialists. Portal, periportal, Zone 1, 2, and 3 regions were prioritized. Following ROI selection, oligos were then UV-cleaved and collected into 96-well plates. Oligos from each ROI were uniquely indexed using Illumina’s i5 x i7 dual-indexing system. Four microliters of a GeoMx DSP sample was used in the PCR reaction. PCR reactions were purified with two rounds of AMPure XP beads (Beckman Coulter) at 1.2 × bead-to-sample ratio. Libraries were paired-end sequenced (2 × 75) on a NovaSeq 6000 sequencer. Serial sections were subjected also to RNA in situ hybridization assay using the RNAScope platform (ACD) and by following the standard vendor protocol.

### NanoString GeoMx DSP data preprocessing

Sequencing reads were compiled into FASTQ files corresponding to each region of interest (ROI) using bcl2fastq. FASTQ files were demultiplexed and converted to Digital Count Conversion (DCC) files with NanoString’s GeoMx NGS DnD Pipeline. The resulting DCC files were converted to an expression count matrix. Raw probe data for 18,372 endogenous genes, with 18,346 genes having one probe per gene and 26 SARS-CoV-2-related genes having 5 probes per gene, as well as 105 global negative probes and 8 SARS-CoV-2 negative probes were generated for 71 ROIs, spanning the portal region, all 3 lobular zones and CD45 regions from 4 patients. The probe counts were normalized using the TMM normalization method implemented in edgeR v.3.28.1. In order to account for unwanted variation, we estimated surrogate variables (SVs) using Iteratively Adaptive Surrogate Variable Analysis (IA-SVA) [117] specifying the model “ ~ Region + Donor”. The expression values were subsequently adjusted with limma’s removeBatchEffect function with Donor as batch and the SVs as covariates.

### Integration of snRNA-seq and DSP data

The data from the nanoString DSP assay were utilized to infer the location of the clusters identified in the snRNA-seq data using the caret (6.0.90) and RandomForest (4.6.14) packages in R 4.0.1. A random forest classifier was trained to predict whether a sample was located in the lobule or in the portal area using pathway activity scores (PAS) as features. The top 200 differentially activated pathways between portal and lobule (100 most upregulated and 100 most downregulated) identified in the nanoString GeoMx DSP data were incorporated as features in the classifier. PAS were estimated, corrected for batch effects, scaled, and centered after summing the nuclei per sample in each cluster. For training, clusters which could be assigned to the lobular or portal area after expert curation were utilized, such as hepatocyte clusters in the lobule and cholangiocytes (BECs) in the portal area. Identified clusters were pseudobulked to reduce noise, and class imbalance was resolved using SMOTE [123], owing to the fact that lobular hepatocyte cells significantly outnumbered portal cells. The samples were split into an 80% training set (224 lobular and 168 portal) and a 20% testing set (30 lobular and 13 portal). Optimal training parameters were identified using fivefold cross validation on the training set through the caret package, resulting in an area under the curve (AUC) of 0.984. Then, the classifier was applied to the remaining clusters. Utilizing SMOTE to address class imbalance, similar results were obtained at the single cell level (Training and CV set: 6944 Lobular and 5208 Portal cells after upsampling, Testing Set: 10,778 Lobular and 434 Portal, resulting in an AUC of 0.998).

### NanoString GeoMx DSP pathway activity scores

As in the case of pathway activity scores for snRNAseq data, a similar approach was utilized for GeoMx DSP datasets. First, ranks were established based on the raw probe counts for each ROI. Then, the ranks were centered and scaled (per ROI). The pathway score was calculated as the sum of the scaled and centered ranks of the genes in the pathway annotation scaled by the square root of the number of genes in the pathway. Unwanted technical variation was accounted for in the pathways scores by estimating surrogate variables (SVs) using the IA-SVA method with the model “ ~ Region + Donor + log(Nuclei Counts) + log(ROI size)”. Then, the pathway scores were adjusted with limma’s removeBatchEffect function with Donor as batch, the SVs, log(Nuclei Counts), and log(ROI size) as covariates.

### NanoString GeoMx DSP viral scores

A SARS-CoV-2 viral score was calculated for the GeoMx DSP WTA ROIs using the extended SARS-COV-2 probe set. In particular, the probes for the S and ORF1ab SARS-CoV-2 genes were utilized. First, the ranks per ROI were calculated based on the raw counts for both the target and negative probes in the SARS-COV-2 probe set, and subsequently centered and scaled. Following a similar approach to the pathway activity scores, the viral score was calculated as the sum of the scaled and centered ranks for the S and ORF1ab probes multiplied by the square root of 2 (the number of genes in the set). Then, the negative and target probe labels were permuted 10,000 times and the viral score was calculated for each permutation to estimate the mean and standard deviation of the viral score. Using these estimates, the observed viral score in each ROI was centered and scaled. Limma’s removeBatchEffect function with the model “log(Nuclei counts) + log(ROI size)” as covariates was utilized to account for ROI size and nuclei counts within the ROI. Finally, the adjusted viral scores were fit to the linear model “ ~ 0 + Donor” using limma to compare the viral scores between donors. For each donor, a contrast was fit to compare the mean adjusted viral score with the mean of the other donors. For example, the contrast for donor L1 is “Donor L1 − (Donor L2 + Donor L3 + Donor L4)/3”.

### NanoString GeoMx DSP differential expression analysis

Limma-trend was utilized to perform differential expression analysis with the GeoMx DSP data. First, batch-corrected expression was fit into the model “ ~ Region” with the limma-trend method and a robust empirical Bayes procedure. Contrasts were utilized to compare the mean of a region against all others, with a gene considered as a region-specific marker if the contrast was significant at an FDR of 0.05 and the region coefficient higher than all other regions. Limma was also used to fit the same model “ ~ Region” on the pathway scores but without the mean–variance trend since the pathway scores are approximately normally distributed. The criteria to select pathway markers were the same as for genes.

For the rotation/scale normalized zonation gradient, ROIs were grouped by lobule and the distance to the zone 1 ROI was calculated per ROI, per lobule. Distances were normalized to be in the [0,1] range. Using the normalized distances, the model “ ~ Normalized Distance” was fit with the batch corrected values, the limma-trend method, and a robust empirical Bayes procedure. We used the coefficient for the normalized distance to identify genes that have an increasing and decreasing pattern across the zonation gradient. For the pathway scores, the same model was fit without the mean–variance trend.

### NanoString CosMx Molecular Imager sample preparation and data analysis

A 5-μm section from a biobanked control liver sample was profiled using the CosMx spatial molecular imager as described in He et al. [124]. In brief, the sample was mounted to a histological slide, and a flow cell for reagent administration was affixed to the slide. The panel was profiled with the 1000-plex Universal Cell Characterization panel and was imaged with stains for PanCK (blue channel), CK8/18 (green), CD45 (red), a cytoplasmic membrane cocktail (yellow), and DAPI (UV). Reagents were flowed across the slide by the CosMx machine to cyclically image each of the targets in the panel, which were decoded based on their assigned barcode sequences and localization as described previously. Segmentation was performed using a consensus model of DAPI channel alone and the composite projection of all other stains. Transcripts were assigned to cells based on the consensus model, and cells with less than 20 transcripts were removed from analysis.

### NanoString CosMx cell annotation

InSituType [125] was used to match healthy liver data from scRNA-seq and snRNA-seq to CosMx data in a supervised manner. Briefly, annotations from [16] were collapsed into 5 major cell types: hepatocytes, immune cells, endothelial cells, mesenchymal cells, and biliary endothelial cells. Then, reference profiles were calculated by aggregating expression profiles. To initially inform the posterior distributions for cell classification, cells were cohorted using fluorescent markers (PanCK, CD45, DAPI), after Gaussian transformation. Background was estimated using the negative probes.

### NanoString CosMx reference integration

The COVID-19 liver nuclei passing QC and not marked as doublets were integrated with the control liver CosMx dataset. First, cells with at least 50 total counts and at least 50 probes with non-zero counts were selected. Subsequently, the CosMx data were subsetted based on the InSituType labels into major cell types. For each cell type, probes with non-zero counts in at least 15% of the cells were selected. The COVID-19 liver nuclei from the matching cell type were integrated with the CosMx liver data into a single expression matrix, normalized to TP100K, and log transformed (log(TP100K + 1)). Following scaling and centering of the log normalized expression values, the first 30 principal components (PCs) were computed and corrected for platform (CosMx and snRNA-seq), and then for batch (each sample as a separate batch) using Harmony. Finally, the shared nearest neighbor graph (SNN) using the Harmony corrected embedding was calculated and utilized to match each cell from the CosMX data set to the closest nuclei from the COVID-19 liver for cluster label assignment.

### Quantitative RT-PCR against SARS-CoV-2

Total RNA was extracted from liver tissue samples using a QIAcube HT (Qiagen) and RNeasy 96 QIAcube HT Kit (Qiagen). RNA was reverse transcribed into cDNA with superscript VILO (Invitrogen). SARS-CoV-2 N (nucleocapsid) gene was cloned into a pcDNA3.1 expression plasmid and transcribed using an AmpliCap-Max T7 High Yield Message Maker Kit (Cellscript) to be utilized as a standard. qPCR was performed in duplicates using a QuantStudio 6 Flex Real-Time PCR System (Applied Biosystems). Viral load was calculated as RNA copies per microgram of total RNA, with a quantitative assay sensitivity of 50 copies. Primers utilized for SARS CoV-2 N genes were:

2019-nCoV_N1-Forward: 5′-GACCCCAAAATCAGCGAAAT-3′, 2019-nCoV_N1-Reverse: 5′-TCTGGTTACTGCCAGTTGAATCTG-3′, and 2019-nCoV_N1-Probe: 5′-FAM-ACCCCGCATTACGTTTGGTGGACC-BHQ1-3′.

### Subgenomic mRNA assay

SARS-CoV-2 E gene subgenomic mRNA (sgmRNA) was assessed by RT-PCR as in Wölfel et al. [40]. A Taqman custom gene expression assay (Thermo Fisher Scientific) was utilized to target the E gene sgmRNA [40]. Standard curves were used to calculate sgmRNA in copies per microgram of total RNA with an assay sensitivity of 50 copies.

### RNAScope

RNA in situ hybridization (ISH) was performed with the RNAScope Multiplex Fluorescent Kit (ACDBio, Newark, CA). All three probes (Hs-TMPRSS2, Hs-ACE2-C2, V-nCoV2019-S-C3) were designed by ACDBio to ensure target specificity. FFPE liver biopsy sections at 5 µm were first de-paraffinized using xylene and ethanol, and incubated in the pretreatment buffer with protease and incubated in a HybEZ oven (ACDBio). The staining of mRNA was achieved by hybridization with the target probes over the pretreated liver tissue, followed by sequential treatment of amplification reagents provided in the RNAScope kit. Each section was dehydrated before being mounted with Pertex (ACDBio). A probe against a housekeeping gene PPIB was used as a positive control (ACDBio).

### Histology, immunohistochemistry, and special tissue staining

Connective tissue stain (Sirius red) and immunohistochemistry (IHC) were performed using formalin-fixed, paraffin-embedded liver biopsy of four COVID-19 patients. For Sirius red staining, liver sections were dewaxed, rehydrated, and stained for 2 min with hematoxylin, then 30 min with a picrosirius red solution (ab246832, Abcam). For IHC staining, antigen retrieval of dewaxed and rehydrated paraffin-embedded liver sections was performed using sodium citrate pH = 6 for α-SMA and pepsin for CK19, respectively, followed by blocking with 10% goat serum for 30 min, and incubation with anti-α-SMA (Cell Signaling Technology, 19,245, 1:400) and anti-CK19 (Sigma-Aldrich, MAB3238, 1:100) primary antibody overnight at 4 °C. After incubation with biotinylated secondary antibody for 1.5 h, detection was performed with the Vectastatin Elite ABC-HRP kit (Vector Laboratories, SP-6100) with the DAB Peroxidase Substrate kit (Vector Laboratories, SK-4100), followed by counterstaining with hematoxylin.

## Supplementary Information

Additional file 1: Supplementary tables. Table S1. Liver serum markers for COVID-19 and control liver samples. Table S2. NanoString GeoMx Digital Spatial Profiling (DSP) Whole Transcriptome Atlas (WTA) SARS-CoV-2 additional probe set. S3-Markers. Table S3. Significant genes and pathways following the zonation gradient in the GeoMx DSP WTA data. Table S4. Differentially expressed genes and pathways for each region of interest in the GeoMx DSP WTA data. Table S5. Differential abundance results comparing COVID with Control livers using a Binomial generalized linear mixed model (GLMM). Table S6. Summary of viral loads using RT-PCR and subgenomic mRNA assay in donors L1-5 who expired due to COVID-19 (LOQ: limit of quantification). Table S7. Association between clinical covariates and donor viral enrichment score. The association tests (Spearman correlation, Kendall’s tau and Wilcoxon rank sum test) were conducted for all samples from all medical centers (All. statistics, All. pvalue) as well as for the samples from medical center A separately (CentrerA.statistic, CenterA.pvalue). Table S8. Summary of liver histopathology findings for samples L1 to L4. H&E staining, CK19, and α-SMA IHC, as well as connective tissue staining (picrosirius red) were performed in four consecutive core biopsies samples and evaluated by an expert clinical liver pathologist (I.N.). Table S9. Curated pathway annotations and signatures used to estimate pathway activity scores.

Additional file 2: Supplementary figures. Figure S1-S8.

Additional file 3: Cluster dictionary.

Additional file 4. Review history.

## Data Availability

Processed sequencing data are available in the Gene Expression Omnibus (GEO, https://www.ncbi.nlm.nih.gov/geo/) under accession number GSE171668 [126], in Zenodo (https://zenodo.org/records/14541192) [127], and raw human sequencing data are available in the controlled access repository DUOS (https://duos.broadinstitute.org/), under dataset IDs DUOS-000126 and DUOS-000127 [128]. The raw sequencing data are available under controlled access, and a DAR (Data Access Request) must be submitted in order to access them. Please refer to the Data Repository Access document on https://github.com/ivlachos/COVID19-Liver for instructions how to access DUOS and how to request data access. Code for the analyses in this article is available on GitHub (https://github.com/ivlachos/COVID19-Liver) [129] and Zenodo (https://zenodo.org/records/14547678, DOI: 10.5281/zenodo.14547677) [130]. The source code is released under the MIT license.
