# Supplementary material for: A single-nucleus and spatial transcriptomic atlas of the COVID-19 liver reveals topological, functional, and regenerative organ disruption in patients
Source: Genome Biol. 2025 Mar 14;26:56. doi: 10.1186/s13059-025-03499-5 (PMC11907808; doi:10.1186/s13059-025-03499-5)
Supplement: Supplementary file 2 — Additional file 2: Supplementary figures. Figure S1-S8. [file 13059_2025_3499_MOESM2_ESM.docx]

**Supplementary Figures**

**
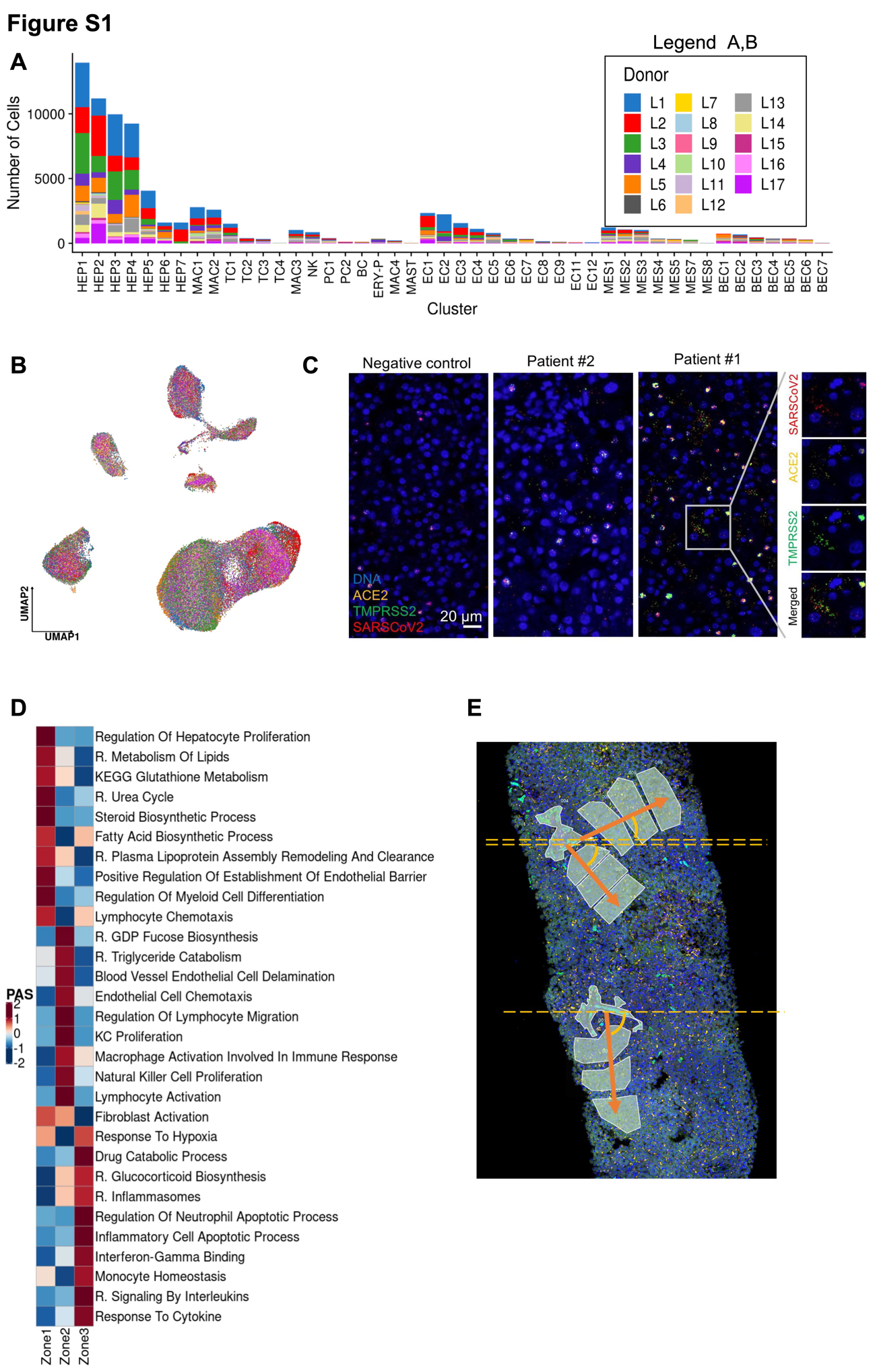
**

**Figure S1**: (A) Number of cells per donor for each cluster. Donors are marked with distinct colors. (B) Uniform manifold approximation and projection (UMAP) of all COVID-19 patient liver cells colored per donor. (C) Liver biopsy tissue (5 μm section) from Donors L1, 2 and a control sample processed with the RNAScope Fluorescent Multiplex Assay (Biotechne). Green: TMPRSS2 (Hs-TMPRSS2); Yellow: ACE2 (Hs-ACE2-C2); Red: SARS-CoV-2 (V-nCoV2019-S-C3); Blue: DAPI. Magnified panels (right) show the single channel staining of each probe in Donor L1. Scale bar represents 20 μm. (D) Heatmap of pathways exhibiting a zonated activity gradient in the DSP WTA data. The zonated pathways were determined by regressing the normalized distance to the zone 1 ROI with the pathway activity score. Color denotes the average pathway activity score of all regions of interest (ROIs) collected for each lobular zone following normalization for batch. Displayed pathways are derived from GO, Biocarta (B), and Reactome (R). (E) Zonation distance diagram depicting the rotation/scale invariant modeling applied for the calculation of the pathway activity score gradient. The ROIs were grouped by lobule and the distance to the corresponding zone 1 ROI was normalized to the (0,1) range. The normalized distance accounts for differences in scale and orientation.

**
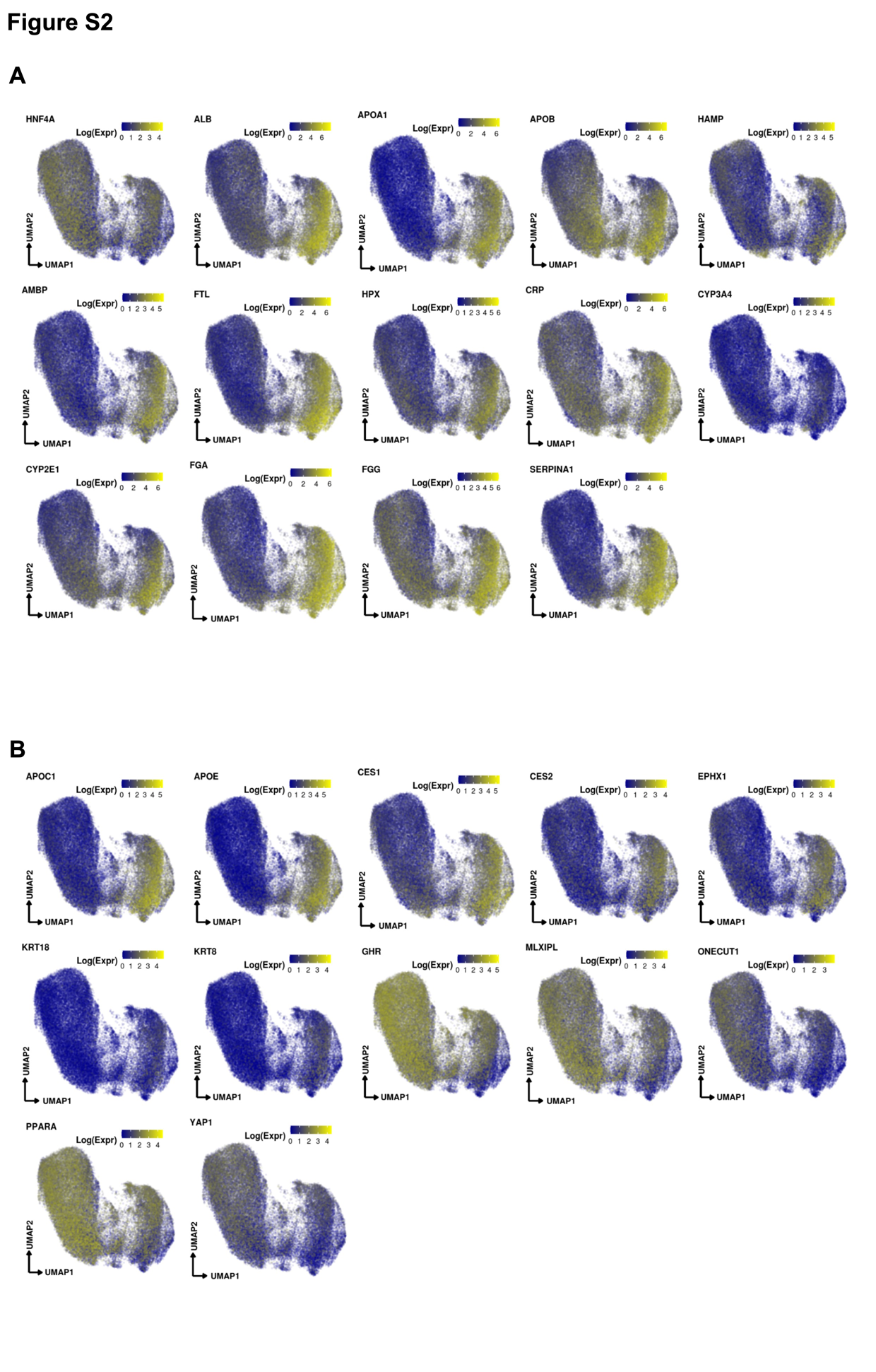
**

**Figure S2**: (A) Uniform manifold approximation and projection (UMAP) depicting gene marker expression in the Hepatocytes compartment. (B) UMAP of markers with higher expression in the left or right portions of the Hepatocyte compartment, denoting a potential division of labor.

**
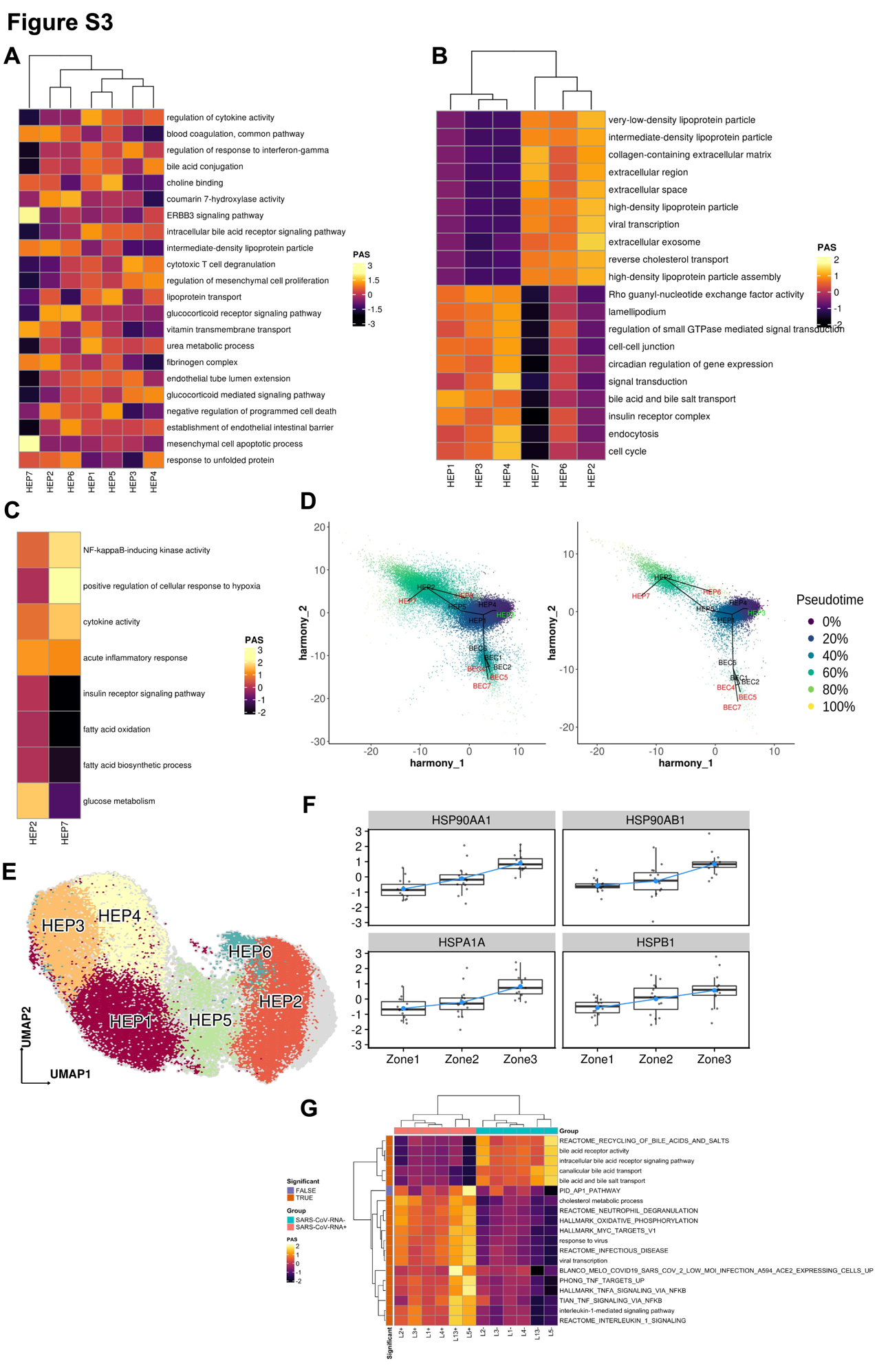
**

**Figure S3**: (A-C) Heatmaps capturing highly active pathways based on pathway activity scores in (A) Hepatocytes, (B) Right versus Left Hepatocyte compartment cellular populations and (C) HEP2 versus HEP7 cellular populations. (D) Pseudotime analysis using Slingshot. Cells are colored based on pseudotime values and are projected on the first 2 primary harmony embeddings across 5 lineages of Hepatocyte and Biliary epithelial cells for (Left) COVID-19 and (Right) healthy liver samples. The initiating and terminal lineage nodes are represented with green and red, respectively. (E) Projection of the CosMx Control Liver hepatocytes onto the COVID-19 hepatocyte UMAP. The HEP7 area of the UMAP is marked with grey, showing a lack of mapped cells in this region. (F) Scaled and centered expression of the *HSP90AA1*, *HS90AB1*, *HSPA1A*, and *HSPB1* genes encoding for Hsp90, Hsp90-beta, Hsp72, and Hsp27 heat shock proteins across the three hepatic lobule zones from the COVID-19 patient DSP WTA spatial transcriptomic dataset. (G) Heatmap capturing pathways based on pathway activity scores in SARS-CoV-2+ hepatocytes compared to SARS-CoV-2- hepatocytes with significant differences (FDR < 0.05).

**
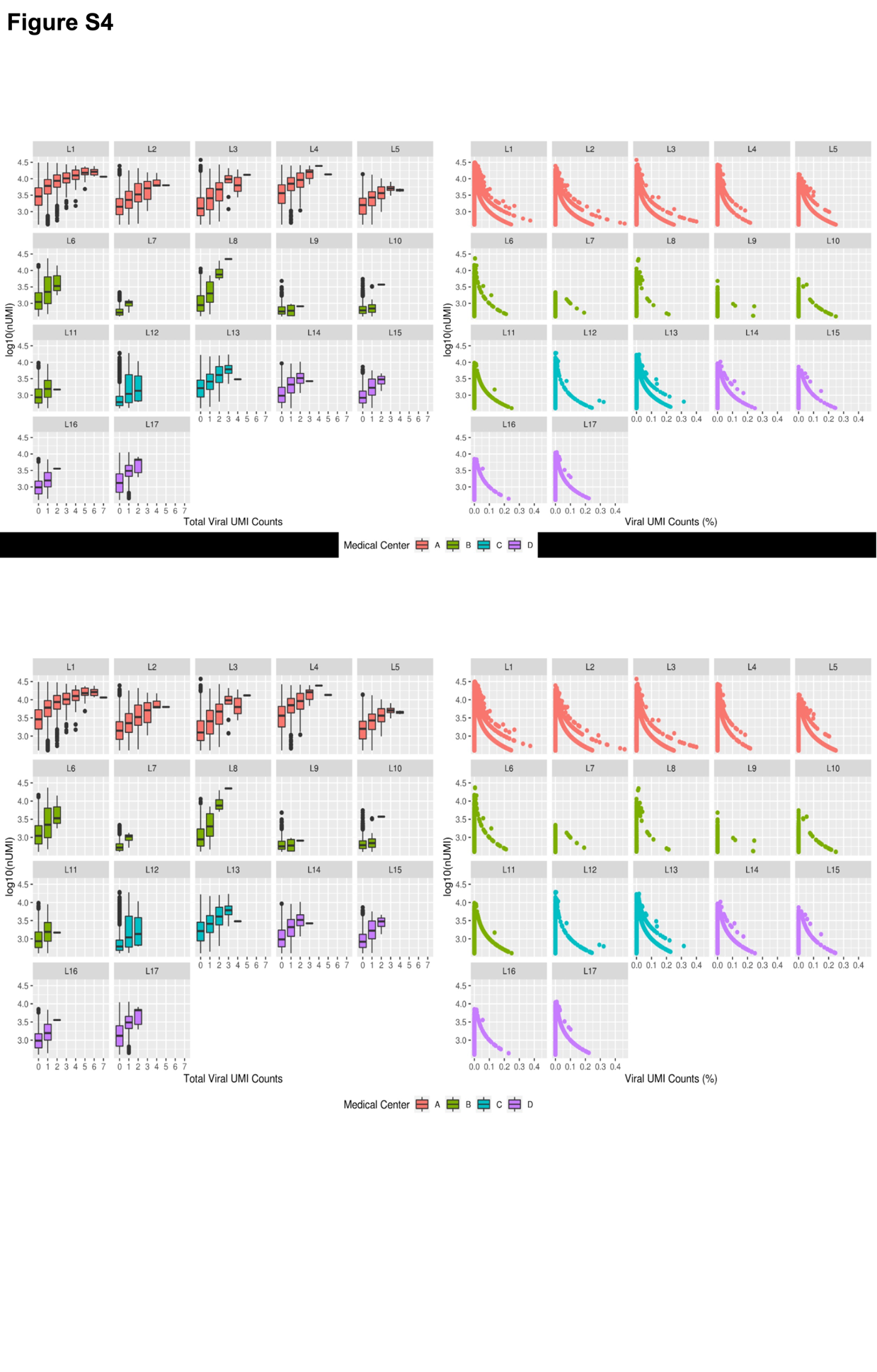
**

**Figure S4:** Viral UMI counts as a function of the number of genes (nGenes) or total UMIs (nUMI) detected in the snRNA-seq data across all donors. Left: Boxplots per viral UMI count depicting the number of detected genes (top) or total UMIs (bottom) on a log10 scale. Right: Scatterplots of % viral UMI counts per cell vs the number of detected genes (top) or total UMIs (bottom) on a log10 scale.

**
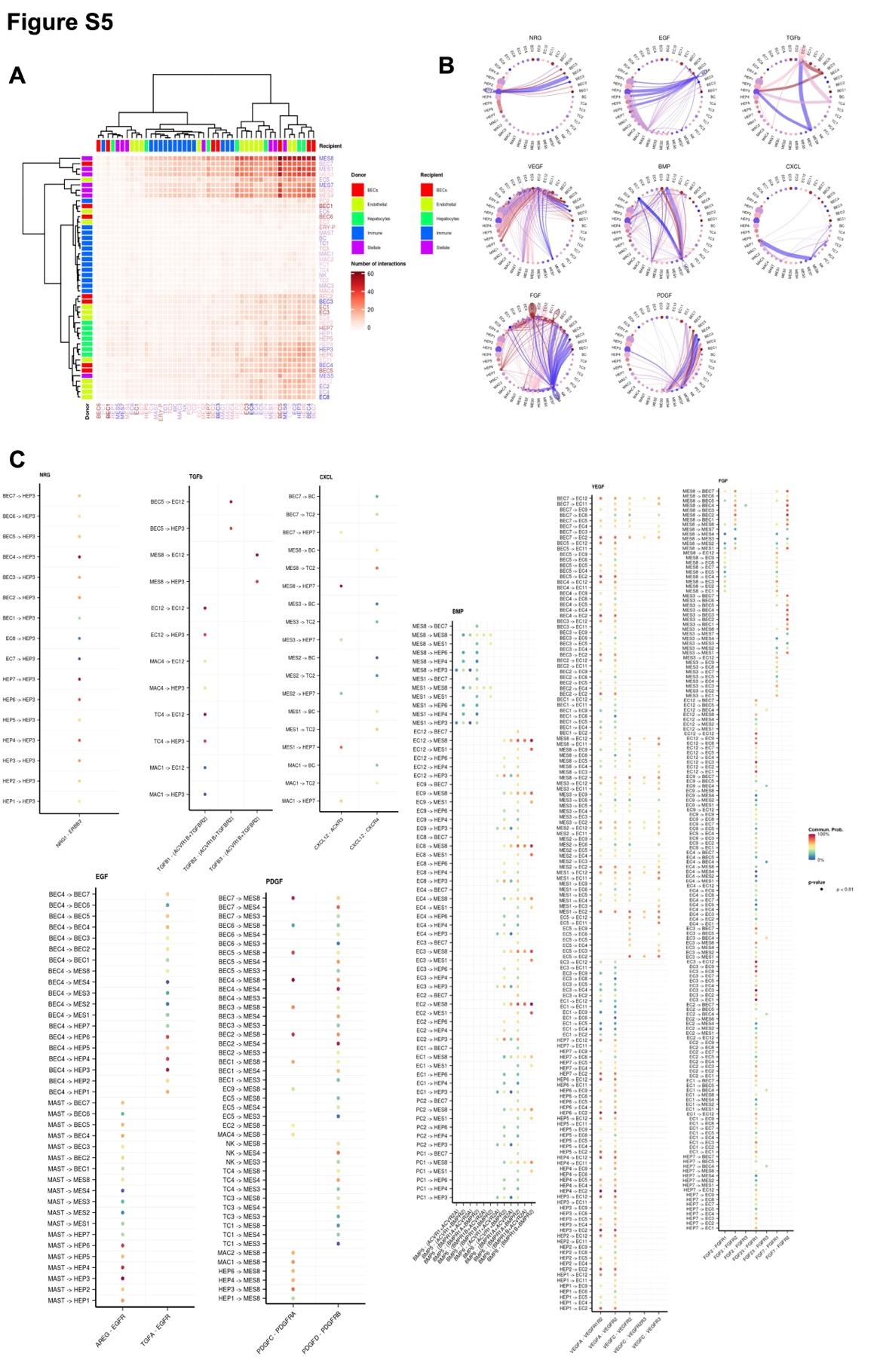
**

**Figure S5:** (A) Heatmap portraying cell-cell communication between the detected cell clusters. The color gradient indicates the number of interactions identified between any two cell groups. Recipient/Donor cell-type color is portrayed in a blue (healthy) to red (COVID-19) gradient, concordantly with the cell composition fold-change differences between healthy and COVID-19 liver samples. (B) Circle plots portraying the aggregated cell-cell communication network in NRG, EGF, TGFb, VEGF, BMP, CXCL, FGF, and PDGF pathways. A thicker edge line indicates a stronger signal, while circle sizes are proportional to the number of cells in each cellular compartment. Donor edge-line and circle color are portrayed in a blue (healthy) to red (COVID-19) gradient, concordantly with the cell composition fold-change differences between healthy and COVID-19 liver samples. (C) Dot plots depicting the relative communication probability of each ligand-receptor (x-axis) in any two significantly interacting cellular compartments (y-axis) (*P-value* < 0.01) for NRG, EGF, TGFb, CXCL, BMP, VEGF, FGF, and PDGF pathways.  Lowest to highest relative communication probability is portrayed with a blue to red color gradient.

**
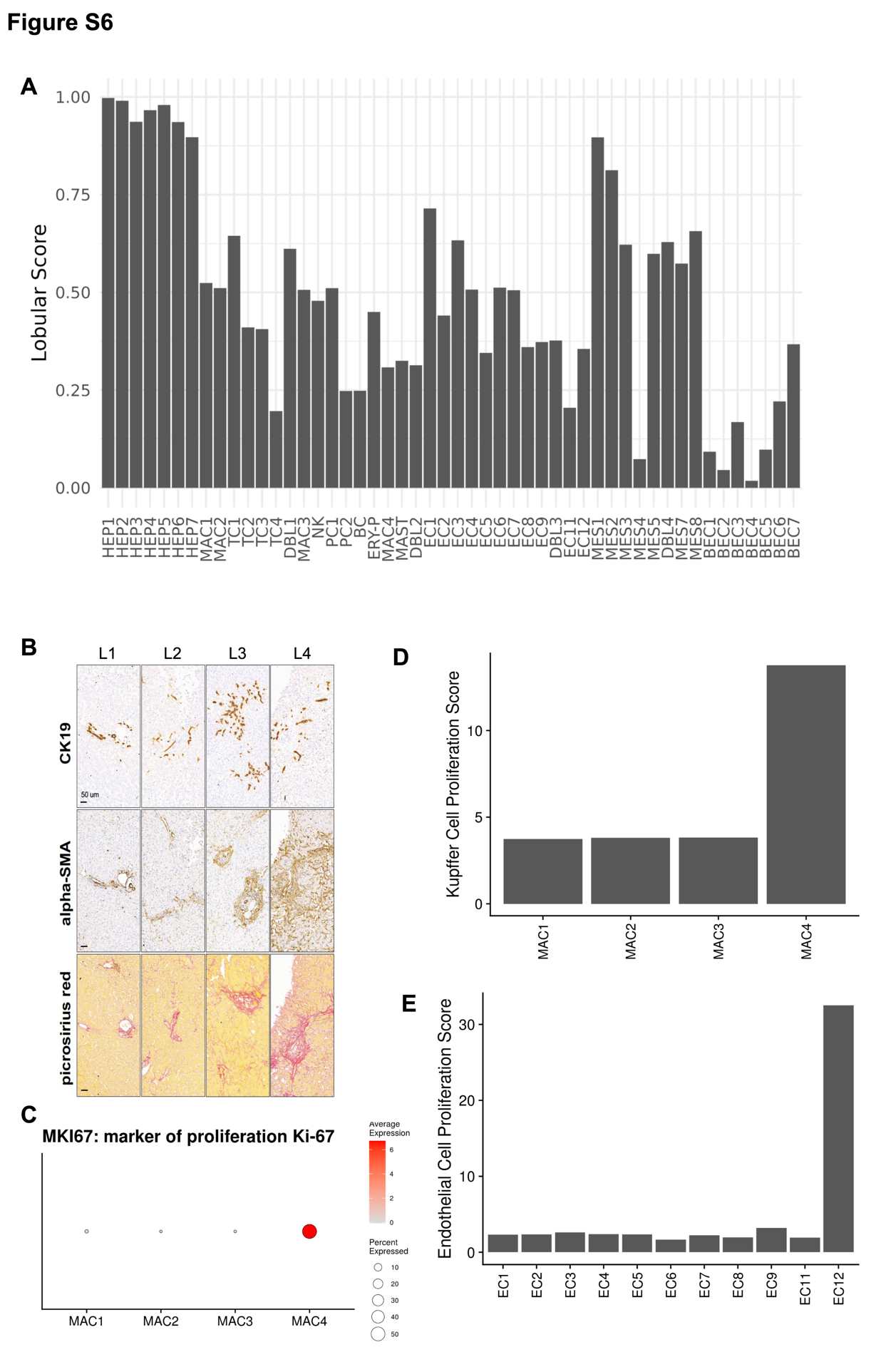
**

**Figure S6:** (A) Probability of each snRNA-seq cluster being localized in the hepatic lobular region based on the DSP WTA data. (B) Representative images of serial sections from four consecutive liver core biopsies samples (BIDMC cohort, donors L1 to L4 as indicated on each column) stained for the ductular/cholangiocyte cell marker CK19, HSC activation maker α-SMA, and connective tissue (picrosirius red), as indicated. All images were acquired at the same magnification (scale bar is 50um). (C) Dot plot for *MKI67* (marker of proliferation *Ki-67*), a canonical marker for proliferation across the macrophage clusters. We characterized MAC4 as Replicating Kupffner Cells. (D) Pathway activity score for the Kupffer cell proliferation signature for the macrophage clusters of the immune compartment. MAC4 was characterized as Replicating Kupffer Cells. (E) Pathway activity score for the endothelial cell proliferation signature for the Endothelial cell clusters. EC12 was annotated as Replicating Endothelial Cells.

**
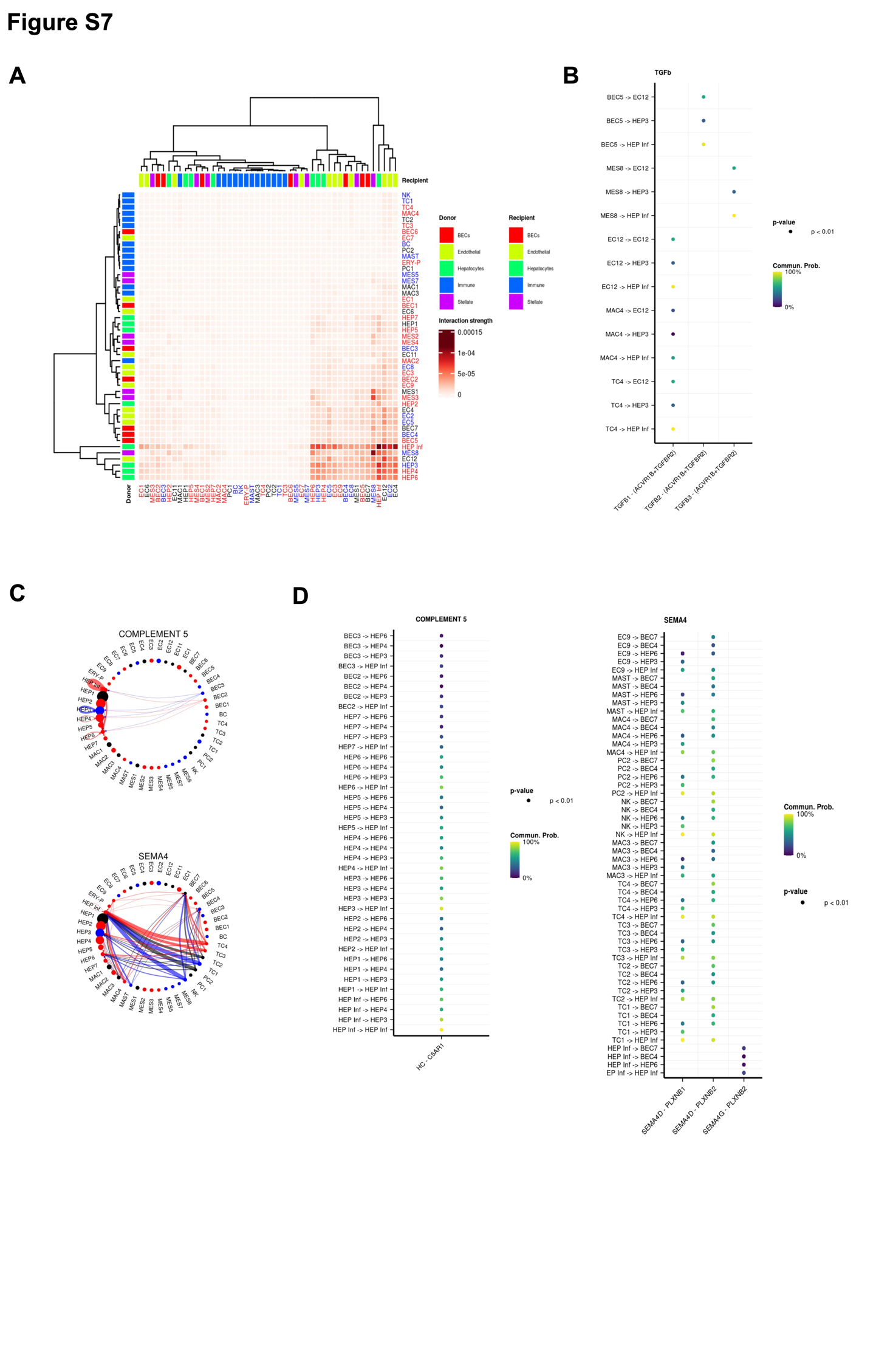
**

**Figure S7:** (A) Heatmap portraying cell-cell communication between the COVID-19 liver cell populations and the enriched hepatocytes in SARS-CoV-2 reads (HEP Inf). The color gradient indicates the strength of interaction between any two cell groups. Recipient/Donor cell-type color is portrayed in blue (significantly increased in healthy), red (significantly increased in COVID-19) and black (no significant difference in cell proportions), concordantly with the cell composition fold-change differences between healthy and COVID-19 liver samples. (B) Dot plot depicting the relative communication probability of each ligand-receptor (x-axis) in any two significantly interacting cellular compartments (y-axis) (*P-value* < 0.01) for the TGFb pathway between HEP Inf and other cell types. Lowest to highest relative communication probability is portrayed with a blue to yellow color gradient. (C) Circle plots portraying the aggregated cell-cell communication network in Complement 5 and SEMA4 pathways between HEP Inf and other cell types. A thicker edge line indicates a stronger signal, while circle sizes are proportional to the number of cells in each cellular compartment. Donor edge-line and circle color are portrayed in blue (significantly increased in healthy), red (significantly increased in COVID-19) and black (no significant difference in cell proportions), concordantly with the cell composition fold-change differences between healthy and COVID-19 liver samples. (D) Dot plot depicting the relative communication probability of each ligand-receptor (x-axis) in any two significantly interacting cellular compartments (y-axis) (*P-value* < 0.01) for Complement 5 and SEMA4 pathways among HEP Inf and other cell types. Lowest to highest relative communication probability is portrayed with a blue to yellow color gradient.

**
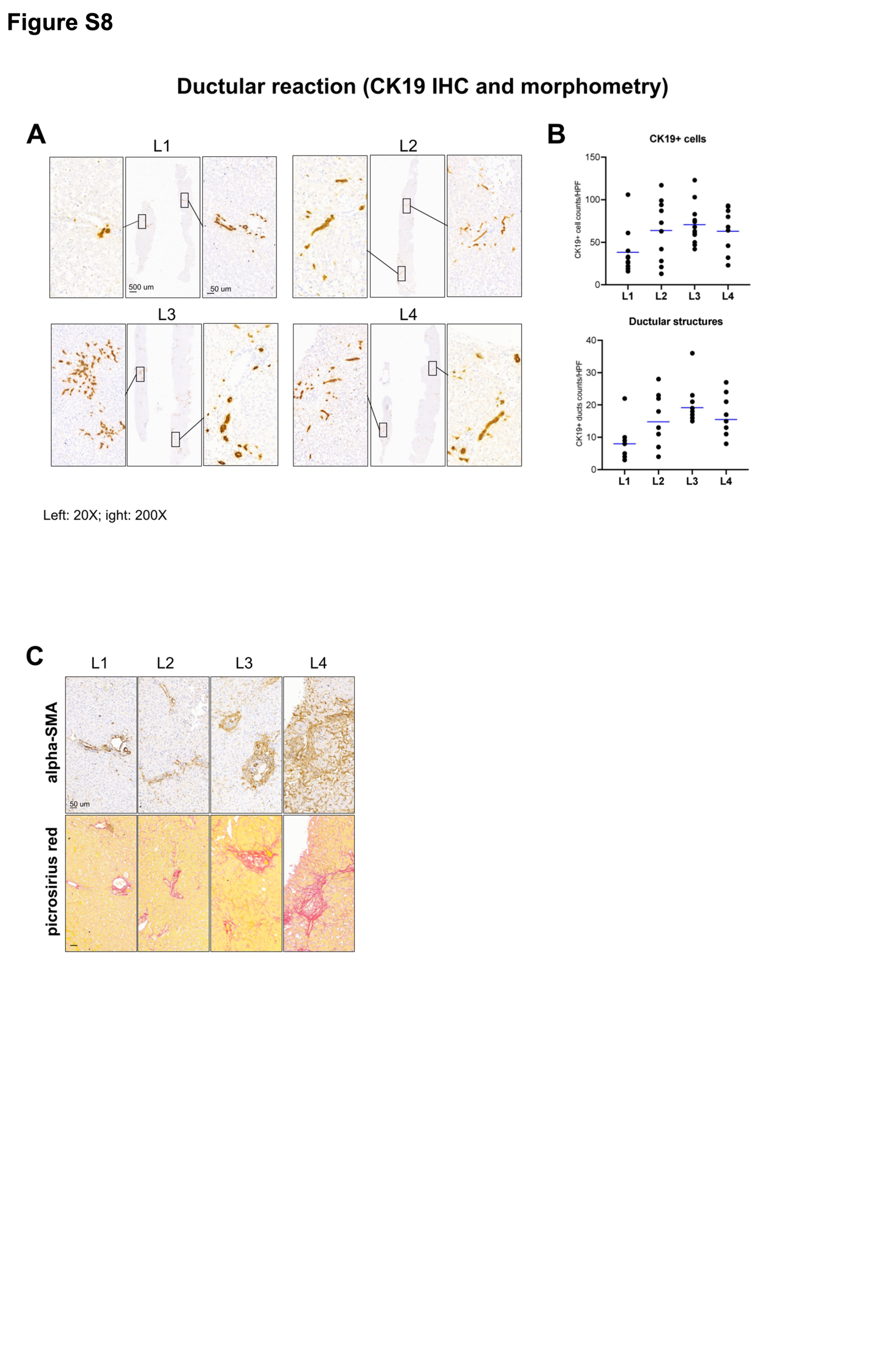
**

**Figure S8:** Pronounced ductular reaction, activation of hepatic stellate cells and fibrosis in livers of deceased patients with severe COVID-19. Representative images of serial sections from four consecutive liver core biopsies samples (BIDMC cohort, donors L1 to L4 as indicated on each column). Cytokeratin 19 immunostaining for (A) ductular reaction marker CK19. Entire core biopsy and representative blow-up images of two portal tract areas shown. Morphometric analysis for CK19+ cell counts and number of ductular structures (B) was performed by counting at least 10 randomly chosen 10 portal tracts per case (high power field, HPF) at 200x magnification. Bar indicates average counts per sample. Representative images of (C) HSC activation marker α-SMA immunohistochemistry, and (C) connective tissue (picrosirius red, fibrillar collagen stained red). Original magnification, 20x (blow-up panel, x200). Scale bar as indicated.
