# Supplementary material for: A single-nucleus and spatial transcriptomic atlas of the COVID-19 liver reveals topological, functional, and regenerative organ disruption in patients
Source: Genome Biol. 2025 Mar 14;26:56. doi: 10.1186/s13059-025-03499-5 (PMC11907808; doi:10.1186/s13059-025-03499-5)
Supplement: Supplementary file 4 — Additional file 4. Review history. [file 13059_2025_3499_MOESM4_ESM.docx]

Review History

**First round of review**

**Reviewer 1**

**Were you able to assess all statistics in the manuscript, including the appropriateness of statistical tests used?**

No, I do not feel adequately qualified to assess the statistics.

**Comments to author:**

The authors of this paper conducted a single-nucleus RNA sequencing and spatial transcriptomic analysis of liver samples from 17 deceased COVID-19 patients. They aimed to understand the molecular mechanisms underlying liver dysfunction in severe COVID-19 and its potential long-term effects. Overall, this study provides several interesting insights into the liver pathology and physiology in COVID-19. However, there are some major concerns:

1、All specimens were post-mortem liver tissues without clear indication of the time of death and sampling time, which may not accurately reflect the impact of COVID-19 on liver tissue.
2、The author claims that "Most patients had multiorgan failure at the time of death, however no patient had clinical or laboratory signs of liver failure or ongoing liver injury", which suggests liver injury of these patients are not severe. Moreover, this is a cross-sectional study，which may not be appropriate to use them to explain the pathogenesis of long COVID.
3、Due to the limitations of the method, a large number of liver parenchymal cells may be obtained, while the proportion of immune cells may be low, leading to potential bias in subsequent analysis.
4、Most of the data are based on bioinformatics analysis, lacking in vivo and in vitro experimental validation, and the lack of intervention experiments to establish causal relationships.
5、One suggestion for improvement would be to include a larger sample size in future studies. Although the authors analyzed samples from 17 COVID-19 decedents, a larger cohort with long-term follow-up would provide more robust and generalizable results. Additionally, it would be beneficial to investigate the long-term effects of COVID-19 on liver function and whether the observed changes persist or resolve over time.

**Reviewer 2**

**Were you able to assess all statistics in the manuscript, including the appropriateness of statistical tests used?**

No, I do not feel adequately qualified to assess the statistics.

**Comments to author:**

The authors performed single nuclei sequencing on 17 liver samples from COVID-19-infected patients and 4 healthy samples. The authors continued with an in-depth spatial analysis on 4 of those. This study provides crucial insights into transcriptome changes and spatially-resolved tissue re-organization in patients with "long COVID-19" infection. Importantly, according to the authors none of the 17 selected patients exhibited signs of ongoing liver injury in their serum values.

Please note that the reviewer does not have the expertise to evaluate all aspects of the bioinformatics used in this manuscript.


MAJOR COMMENTS

On one hand, this study generates a substantial amount of data that will be of high interest for future studies and must be shared with the global research community. On the other hand, one might expect the authors to further discuss their preliminary hypotheses on molecular mechanisms that could drive a COVID-19-related liver pathology. The manuscript remains very descriptive in the current state, and does not elaborate much on the potential implications of the findings.

The authors describe that the COVID-19-specific HEP7 cells derive from the mature HEP2 population. Could then the authors elaborate on, for instance, the reasons and the functional consequences of this? Also, do HEP7 cells exhibit reduced metabolic functions as compared to HEP2? In COVID-19 versus healthy samples, the proportion of HEP2 is increased in COVID-19 patients as well. Have the authors an explanation for this phenomenon, and how is that to be put in perspective with disease progression?

It is intriguing that viral RNAs are not only detected in HEP7 cells. This would suggest that not all hepatocytes respond to viral infection by drastic trancriptome changes, such as HEP2 transitioning to HEP7. Could the authors elaborate on the potential specificities of HEP2 cells that would make them prone to response to viral infection by e.g., increased acute phase response proteins? Similarly, do the authors have a proposed explanation for e.g., the predisposition of HEP4 cells to show signs of activated NFkB and type 1 interferon signaling after viral infection?

Considering the relative small tissue areas that were analyzed in all samples, the authors might as well discuss some limitations that could be due to sampling artefacts.

The manuscript conclusion states "Our study revealed SARS-CoV-2 RNA positive hepatocytes with an expression phenotype similar to infected lung epithelial cells", whereas the cover letter states "We identified human hepatocytes infected by SARS-CoV-2 and characterized their expression profiles at single cell resolution, which are in many ways distinct from infected lung epithelial cells, reflecting the combined viral and systemic effects of COVID-19 on hepatocyte populations.". May the authors clarify these? Furthermore, this point is not explained enough in the manuscript. What would be the distinct/resembling aspects between infected lung and hepatocytes?

Fig. S6B is moderately convincing to make the point of varying degrees of ductular reaciton between L1 to L4. Additional fields of view and quantitations would be more appropriate.


MINOR COMMENTS

Page 6 line 19: Protein names should not be italicized.

Page 17 line 48 did you mean "liver fibrosis"?

Ductular reaction and other histopathology findings have been characterized by a pathologist in only 4 liver samples (L1 to L4). Why were those samples chosen for in-depth analysis? Also, livers 5, 12 and 13 are from patients with a high total bilirubin levels in the blood. How could that affect the findings? Could additional information, such as fibrosis and inflammation scores, be provided for all patients?

**Authors Response**

**Point-by-point responses to the reviewers’ comments:**

***Reviewer #1:***  *The authors of this paper conducted a single-nucleus RNA sequencing and spatial transcriptomic analysis of liver samples from 17 deceased COVID-19 patients. They aimed to understand the molecular mechanisms underlying liver dysfunction in severe COVID-19 and its potential long-term effects. Overall, this study provides several interesting insights into the liver pathology and physiology in COVID-19. However, there are some major concerns:*

We would like to thank the Reviewer for their comments and for noting the interesting insights uncovered by this study. We hope that our point-by-point response addresses all provided comments.

*1. All specimens were post-mortem liver tissues without clear indication of the time of death and sampling time, which may not accurately reflect the impact of COVID-19 on liver tissue.*

We would like to thank the Reviewer for their comment. We agree that such information would be very useful to the Readers. We collected and added information regarding the duration of disease as well as ischemia time for all patients to **Table 1**. Both were prioritized as the key descriptors requested by the Reviewer in place of the actual time of death, since it could be used to de-identify the samples. Reviewing this information, in combination with the findings of the study, one can appreciate that the minimal ischemia time (<24h for all samples) and the viral presence being still identified across different modalities (RNAscope, RT-PCR, NanoString GeoMx DSP) point that these samples are representative of post-acute severe COVID-19 phenotypes, some with still active infection. We also acknowledge that this study set is representative only for patients who died from severe COVID-19 and do not reflect the liver phenotypes of subjects with moderate or less severe disease.

In order to mirror the above information also clearly in the text, the following mentions have been added to the manuscript:

[**Results**]: “All samples were obtained postmortem using either ultrasound-guided needle biopsy or surgical dissection by following stringent protocols established previously [7] (**Additional File 1: Table S1, Methods**).”

[**Discussion**]: “Our study was limited by including a relatively small number of patients (n=17) with a severe post-acute COVID-19 phenotype, not enabling us to directly assess moderate and less severe or acute manifestations of the disease.”

*2. The author claims that "Most patients had multiorgan failure at the time of death, however no patient had clinical or laboratory signs of liver failure or ongoing liver injury", which suggests liver injury of these patients are not severe. Moreover, this is a cross-sectional study which may not be appropriate to use them to explain the pathogenesis of long COVID.*

This is an accurate observation, and we believe that it’s a central point we are making in the study. At the beginning of the pandemic, the first observations of abnormal liver biochemistry in COVID-19 patients brought a lot of attention to a potential liver involvement in severe COVID-19. However, since very few cases were reported with acute liver failure, relevant research efforts dwindled. This is why we believe this study is timely and important.

We also agree with the Reviewer that most patients do not have liver failure or significant liver injury. However, today, COVID-19 is well recognized as a multisystem disorder with well-established liver implications, including abnormal liver biochemistry and, in some cases, post-COVID-19 cholangiopathy. In our detailed investigation, we observe extensive remodeling in numerous cell populations, especially in the endothelial compartment, with significant potential functional implications. Our study demonstrates that subclinical liver injury is common in this multi-center cohort and could be a stepping stone to understanding post-acute COVID-19 liver sequelae.

The inclusion of the healthy control group renders the study a case - control and not a cross-sectional study. However, we agree with the Reviewer, and based on our experience and learnings from this comprehensive investigation, we are planning on designing follow-up studies including additional control groups, such as autopsy samples from patients with long-COVID who succumbed to causes unrelated to COVID-19.

To clarify all the raised issues, in the revised manuscript, we report that:

[**Abstract**]: “Despite the absence of a clinical acute liver injury phenotype, endothelial cell composition was dramatically impacted in COVID-19, concomitantly with extensive alterations and profibrogenic activation of reactive cholangiocytes and mesenchymal cells.”

And that:

[**Discussion**]: “We also found extensive changes in the composition and expression programs of non-parenchymal cells across the liver lobule and portal triad in COVID-19… These cellular and expression changes induced by COVID-19, despite an absence of significant tissue injury, point to subclinical yet profound effects of COVID-19 on the human liver, and may carry long-term health implications for those who recover from acute infection”

**[Discussion]:** “Future studies expanding the current study design could be very useful to identify further phenotypic and mechanistic differences, including liver tissue samples not only from healthy controls and COVID-19 patients but also from long COVID patients who succumbed to causes unrelated to COVID-19, as well as from patients with non-COVID-19-related pneumonia/ARDS.”

*3. Due to the limitations of the method, a large number of liver parenchymal cells may be obtained, while the proportion of immune cells may be low, leading to potential bias in subsequent analysis.*

We would like to stress that the accurate depiction of cellular proportions of examined tissues is actually a major advantage of single-nucleus RNA sequencing vs single-cell RNA sequencing (Koenitzer et al. 2020). In single-cell sequencing, cells with a smaller diameter, such as T cells, are majorly overrepresented. Liver single-cell studies have notoriously suffered from this effect, with many studies having no or a minimal number of hepatocytes being studied (Van Melkebeke et al. 2024). For instance, in the Aizarani, *et al.* study, Nature 2019, out of the 10,683 cells studied from normal liver samples, only 2,534 hepatocytes were included (23.7% of all cells), limiting the ability to capture hepatocyte function in high granularity. Furthermore, all other non-parenchymal cell populations comprised 8,149 cells (Aizarani et al. 2019).

Members of our team in Andrews *et al.* in Hepatology Communications, compared single-cell RNA-seq and single-nuclei-RNA-seq applied to human liver samples (Andrews et al. 2022). They noted that cell type frequencies are distorted in scRNA-Seq data due to the resiliency of immune cells to tissue dissociation compared to parenchymal cells. snRNA-seq enables the detection of many additional cell populations and states, especially of hepatocytes, cholangiocytes, and mesenchymal cells, which are often underrepresented in liver scRNA-seq data.

In this study we generated 80,808 cell profiles passing quality control from 17 patients, with 51,605 hepatocytes, 12,346 immune / blood cells, 9,278 endothelial cells, 4,647 mesenchymal cells, and 2,932 biliary epithelial cells / Cholangiocytes, making it one of the largest single cell/single nucleus study ever conducted from human liver samples. The high number of nuclei for parenchymal and non-parenchymal cells provided us with very high power to identify rare cell populations or perform comparisons. Specifically, 5 populations were identified to be significantly differentially abundant in COVID-19 subjects vs controls in parenchymal cells, while 25+ populations with significant differences were identified in non-parenchymal cell populations

We consider the Reviewer’s comment very important, and the following text has been added to the manuscript:

[**Discussion**]: “In this study, single nucleus sequencing was an advantageous modality, supporting highly standardized and streamlined sample collection in very demanding circumstances as opposed to single-cell sequencing, where fresh samples are required to be readily processed. Furthermore, it supported the generation of a large dataset with >80K nuclei passing QC, with a representation of parenchymal and non-parenchymal cell populations resembling more liver physiology as opposed to single-cell sequencing, where lymphocyte populations are often largely overrepresented”.

**References**

- Aizarani, Nadim, Antonio Saviano, Sagar, Laurent Mailly, Sarah Durand, Josip S. Herman, Patrick Pessaux, Thomas F. Baumert, and Dominic Grün. 2019. “A Human Liver Cell Atlas Reveals Heterogeneity and Epithelial Progenitors.” Nature 572 (7768): 199–204.
- Andrews, Tallulah S., Jawairia Atif, Jeff C. Liu, Catia T. Perciani, Xue-Zhong Ma, Cornelia Thoeni, Michal Slyper, et al. 2022. “Single-Cell, Single-Nucleus, and Spatial RNA Sequencing of the Human Liver Identifies Cholangiocyte and Mesenchymal Heterogeneity.” Hepatology Communications 6 (4): 821–40.
- Koenitzer, Jeffrey R., Haojia Wu, Jeffrey J. Atkinson, Steven L. Brody, and Benjamin D. Humphreys. 2020. “Single-Nucleus RNA-Sequencing Profiling of Mouse Lung. Reduced Dissociation Bias and Improved Rare Cell-Type Detection Compared with Single-Cell RNA Sequencing.” American Journal of Respiratory Cell and Molecular Biology 63 (6): 739–47.
- Van Melkebeke, Lukas, Jef Verbeek, Dora Bihary, Markus Boesch, Bram Boeckx, Rita Feio-Azevedo, Lena Smets, et al. 2024. “Comparison of the Single-Cell and Single-Nucleus Hepatic Myeloid Landscape within Decompensated Cirrhosis Patients.” Frontiers in Immunology 15 (February): 1346520.

*4. Most of the data are based on bioinformatics analysis, lacking in vivo and in vitro experimental validation, and the lack of intervention experiments to establish causal relationships.*

We agree with the Reviewer that this is a limitation of the study design. However, we believe that the provided atlas of the post-acute COVID-19 liver can become the foundation for future mechanistic studies that could lead to targeted therapeutics, diagnostics, or intervention strategies. Single-cell and spatial tissue profiling studies generating such atlases and highly granular comparisons are fueling discovery and innovation for COVID-19

(Delorey et al., Nature 2021; Edahiro et al., Nature Genetics, 2023; Ren et al. Cell, 2021; Feys et al., Lancet, 2024), but also cancer (Reed et al., Nature Genetics, 2024), or even neurodegenerative diseases (Sun et al., Nature Neurosciences, 2023; Mathys et al., Nature, 2019), despite not having downstream *in vitro* or *in vivo* investigations. We believe that most groups, including ours, chose this study design since these single-cell/spatial tissue atlases require an enormous amount of resources and personnel hours to be conducted, which renders downstream investigations the focus of follow-up studies for purely pragmatic reasons. Furthermore, we consider that it is an obligation not only to the research community but also to the patients and their families to communicate this thorough investigation in a timely manner.

To further clarify this matter, the following text has been added to the discussion:

[**Discussion**]: “Importantly, this investigation focused on the generation and in-depth analysis of the single cell and spatial tissue profiling data from the collected tissue samples, on the comparisons between healthy controls and COVID-19 patients, as well as on the transcriptional phenotyping of SARS-CoV-2 positive cells. However, these observations have not been followed by downstream experimental investigations, which is a limitation. Nevertheless, single-cell and spatial-omic profiling studies of patient tissues have extended our understanding of the cell- and tissue-specific effects of COVID-19, by providing a highly granular characterization of the cellular populations, tissue architecture, and interactions in a hypothesis-free manner, offering novel insights and fueling reverse translation [7,83-85]. We hope that the generated data from this atlas of the post-acute severe

COVID-19 liver could enable follow-up mechanistic studies but also the generation of targeted diagnostic and intervention strategies.”

**References**

- Delorey, Toni M., Carly G. K. Ziegler, Graham Heimberg, Rachelly Normand, Yiming Yang, Åsa Segerstolpe, Domenic Abbondanza, et al. 2021. “COVID-19 Tissue Atlases Reveal SARS-CoV-2 Pathology and Cellular Targets.” Nature 595 (7865): 107–13.
- Edahiro, Ryuya, Yuya Shirai, Yusuke Takeshima, Shuhei Sakakibara, Yuta Yamaguchi, Teruaki Murakami, Takayoshi Morita, et al. 2023. “Single-Cell Analyses and Host Genetics Highlight the Role of Innate Immune Cells in COVID-19 Severity.” Nature Genetics 55 (5): 753–67.
- Feys, Simon, Sam Vanmassenhove, Sirima Kraisin, Karen Yu, Cato Jacobs, Bram Boeckx, Seppe Cambier, et al. 2024. “Lower Respiratory Tract Single-Cell RNA Sequencing and Neutrophil Extracellular Trap Profiling of COVID-19-Associated Pulmonary Aspergillosis: A Single Centre, Retrospective, Observational Study.” The Lancet. Microbe 5 (3): e247–60.
- Mathys, Hansruedi, Jose Davila-Velderrain, Zhuyu Peng, Fan Gao, Shahin Mohammadi, Jennie Z. Young, Madhvi Menon, et al. 2019. “Single-Cell Transcriptomic Analysis of Alzheimer’s Disease.” Nature 570 (7761): 332–37.
- Reed, Austin D., Sara Pensa, Adi Steif, Jack Stenning, Daniel J. Kunz, Linsey J. Porter, Kui Hua, et al. 2024. “A Single-Cell Atlas Enables Mapping of Homeostatic Cellular Shifts in the Adult Human Breast.” Nature Genetics 56 (4): 652–62.
- Ren, Xianwen, Wen Wen, Xiaoying Fan, Wenhong Hou, Bin Su, Pengfei Cai, Jiesheng Li, et al. 2021. “COVID-19 Immune Features Revealed by a Large-Scale Single-Cell Transcriptome Atlas.” Cell 184 (23): 5838.
- Sun, Na, Leyla Anne Akay, Mitchell H. Murdock, Yongjin Park, Fabiola Galiana-Melendez, Adele Bubnys, Kyriaki Galani, et al. 2023. “Single-Nucleus Multiregion Transcriptomic Analysis of Brain Vasculature in Alzheimer’s Disease.” Nature Neuroscience 26 (6): 970–82.

*5. One suggestion for improvement would be to include a larger sample size in future studies. Although the authors analyzed samples from 17 COVID-19 decedents, a larger cohort with long-term follow-up would provide more robust and generalizable results. Additionally, it would be beneficial to investigate the long-term effects of COVID-19 on liver function and whether the observed changes persist or resolve over time.*

We agree with the Reviewer’s recommendation. We wanted to stress that access to liver samples from living healthy individuals is exceptionally difficult for long-term follow-up studies due to practical and, most importantly, ethical limitations. However, we understand the important information they could generate. We also agree with the Reviewer that even though this is the largest single-cell study of human liver samples to date (healthy or diseased), it still doesn’t capture the potential heterogeneity on a population scale.

We believe that the aforementioned text accurately captures these limitations:

[**Discussion**]: “Our study was limited by including a relatively small number of patients (n=17) with a severe post-acute COVID-19 phenotype, not enabling us to directly assess moderate and less severe or acute manifestations of the disease. We plan on building upon the generated information with studies comprising a larger and more heterogeneous population.”

[**Discussion**]: “Future studies expanding the current study design could be very useful to identify further phenotypic and mechanistic differences, including liver tissue samples not only from healthy controls and COVID-19 patients but also from long COVID patients who succumbed to causes unrelated to COVID-19, as well as from patients with non-COVID-19-related pneumonia/ARDS.”

***Reviewer #2****: The authors performed single nuclei sequencing on 17 liver samples from COVID-19-infected patients and 4 healthy samples. The authors continued with an in-depth spatial analysis on 4 of those. This study provides crucial insights into transcriptome changes and spatially-resolved tissue re-organization in patients with "long COVID-19" infection. Importantly, according to the authors none of the 17 selected patients exhibited signs of ongoing liver injury in their serum values.*

*Please note that the reviewer does not have the expertise to evaluate all aspects of the bioinformatics used in this manuscript.*

We would like to thank the Reviewer for their positive comments and for noting that the study provides crucial insights.

**MAJOR COMMENTS**

*1. On one hand, this study generates a substantial amount of data that will be of high interest for future studies and must be shared with the global research community. On the other hand, one might expect the authors to further discuss their preliminary hypotheses on molecular mechanisms that could drive a COVID-19-related liver pathology. The manuscript remains very descriptive in the current state, and does not elaborate much on the potential implications of the findings.*

We would like to thank the Reviewer for their comment and for recognizing the importance of sharing this study and generated data with the global research community. Following the Reviewer’s comment, we have significantly expanded the Discussion section, offering hypotheses supported by our domain experts and the literature. We also expanded the Discussion with an additional focus on key mechanistic components, such as cell-cell communication, underpinning COVID-19 liver pathology. We paid attention to elaborate on how these findings can be exploited in further basic and translational research, and to delineate that future downstream investigations *in vitro* and *in vivo* are required to elucidate further these mechanisms.

The added text sections include:

[**Discussion**]: “Meanwhile, profibrogenic/reactive cholangiocytes were identified as characteristic populations expanding in the COVID-19 liver, representing a pathological “ductular reaction” - an extensive remodeling and scarring of biliary compartment, secondary to local as well as systemic liver injury [37]. This striking observation was validated by connective tissue stains and conventional immunohistochemistry and morphometry for CK19+ ducts, clearly showing various degree of ductular reaction and associated a-SMA+ myofibroblast activation, consistent with emerging reports of COVID-19-induced sclerosing cholangitis (fibrotic disease of bile ducts) [77], which in most severe cases may require liver transplantation [78]. Our high-resolution observations and comprehensive molecular characterization of cell-cell interactions in subacute COVID-19 cholangiopathy may provide a unique opportunity to elucidate relevant drivers of other chronic cholangiopathies of enigmatic nature and currently without effective therapy, such as primary sclerosing cholangitis (PSC), which is challenging to study due to slow progression and scarce opportunities to analyze the liver tissue in early disease stages. In this respect, we have identified several mediators (TGFbeta, PDGF) that were well studied in cholangiopathies, such as PSC, as well as those that are much less explored, such as VEGF. Since several VEGF inhibitors were developed, rapid functional and therapeutic validation of this new target is feasible and is an area of our future studies.”

[**Discussion**]: “Our results also recapitulated the observation of high Interferon signaling pathway activity, as were suggested in a bulk RNA-seq analysis of 5 samples from SARS-CoV-2 positive livers compared against 5 SARS-CoV-2 negative liver samples [4]. Elevated proportions of SARS-CoV-2+ hepatocyte populations were also noted, with high abundances of cells in the terminally differentiated states as well as the emergence of a SARS-CoV-2+-specific cluster (HEP7) with a shift in function from metabolic processing to response to infection and inflammation. Terminally differentiated hepatocytes demonstrated both high metabolic activity and an increased inflammatory response, characterized by high expression of acute phase proteins, which may enable the increased response to viral infection pathway activity observed in SARS-CoV-2+ cells of these populations [74]. Additionally, the increased abundance of these cells in COVID-19 patients, along with their elevated metabolic processes, may reflect a compensatory mechanism to maintain liver function and address the increased metabolic demands and detoxification needs during disease progression [31]. On the other hand, viral-infected hepatocytes not included in the COVID-19-specific lineage maintained a highly stressed state and elevated autophagy activity. These populations presented high expression and activity of autophagy-related pathways and genes, which could represent an effort of the cells to induce clearance of viruses through viral component encapsulation in autophagosomes, and lysosomal degradation. However, coronaviruses have been shown to turn autophagy into a double-edged sword, and through its modulation, they can prevent degradation, further enhancing their replication and persistence within host cells [75][76].”

[**Discussion**]: “We also found extensive changes in the composition and expression programs of non-parenchymal cells across the liver lobule and portal triad in COVID-19….These cellular and expression changes induced by COVID-19, despite an absence of significant tissue injury, point to subclinical yet profound effects of COVID-19 on the human liver, and may carry long-term health implications for those who recover from acute infection.”

*2. The authors describe that the COVID-19-specific HEP7 cells derive from the mature HEP2 population. Could then the authors elaborate on, for instance, the reasons and the functional consequences of this? Also, do HEP7 cells exhibit reduced metabolic functions as compared to HEP2? In COVID-19 versus healthy samples, the proportion of HEP2 is increased in COVID-19 patients as well. Have the authors an explanation for this phenomenon, and how is that to be put in perspective with disease progression?*

Following the Reviewer’s insightful comment, we expanded significantly our investigation to shed light on functional differences between the HEP7 and HEP2 populations at the gene and pathway levels. These additional analyses reveal that, indeed, there is a markedly reduced activity of typical metabolic functions in the HEP7 cluster, including a reduction of lipid and glucose metabolism, as compared to HEP2 hepatocytes (***Figure 1***). On the other hand, HEP7 cells highly up-regulated immune response, cellular stress, and hypoxia-related pathways, reflecting a shift in function from metabolic function to the response to infection, inflammation, and cellular stress. We hypothesize that the increase in HEP2 cells is part of a compensatory response, which we also commented on in the Discussion section.

We also expanded our analyses by directly comparing HEP2 cells from COVID-19 liver samples and healthy controls (***Figure 2***). There, we see not only an increase of heat shock protein but also of FGFR signaling pathways with potentially direct implications on glycogen, lipid, and bile acid metabolism. FGFR4 signaling in hepatocytes upregulates glycogen and fatty acid metabolism while suppressing bile acid metabolism (Dolegowska et al. 2019), playing an essential role in systemic lipid, glucose, and bile acid homeostasis (Gadaleta and Moschetta 2019; X. Li et al. 2024; Huang et al. 2007). Similarly, FGFR1 is involved in lipid and glucose metabolism (Yang et al. 2012; K. Li et al. 2012). Heat shock proteins can either promote or inhibit viral infection by regulating immune pathways, the cell cycle, and by directly binding to viral proliferation-related factors (Zhang and Yu 2022). Interestingly, heat shock proteins exhibit a dual role in COVID-19, facilitating viral replication while modulating the host inflammatory response (Kasperkiewicz 2021; Zhang and Yu 2022). Moreover, inhibition of the molecular chaperone heat shock protein 90 (Hsp90) has been shown to limit SARS-CoV-2 infection (Zhao et al. 2023).

**Figure 1.** Heatmap capturing highly active pathways based on pathway activity scores in HEP2 versus HEP7 cellular populations.

**Figure 2**. Heatmap of highly active pathways based on pathway activity scores (PAS, FDR < 0.05) in COVID-19 HEP2 cells as compared to HEP2 cells from healthy liver samples.

Based on these observations, the following have been added to the manuscript:

[**Results**]: Of note, pathways such as fatty acid biosynthesis, insulin signaling, and glucose metabolism were less active in the HEP7 cluster compared to HEP2 (**Additional file 2: Fig. S3c**). HEP7 cells showed significant upregulation of pathways involved in immune responses and cellular stress, reflecting a shift in function from typical metabolic processing to response to infection and inflammation [1].

[**Discussion**]: Elevated proportions of SARS-CoV-2+ hepatocyte populations were also noted, with high abundances of cells in the terminally differentiated states as well as the emergence of a SARS-CoV-2+-specific cluster (HEP7) with a shift in function from metabolic processing to response to infection and inflammation. Terminally differentiated hepatocytes demonstrated both high metabolic activity and an increased inflammatory response, characterized by high expression of acute phase proteins, which may enable the increased response to viral infection pathway activity observed in SARS-CoV-2+ cells of these populations [1]. Additionally, the increased abundance of these cells in COVID-19 patients, along with their elevated metabolic processes, may reflect a compensatory mechanism to maintain liver function and address the increased metabolic demands and detoxification needs during disease progression [2].

**References**

- Dolegowska, Katarzyna, Malgorzata Marchelek-Mysliwiec, Monika Nowosiad-Magda, Michal Slawinski, and Barbara Dolegowska. 2019. “FGF19 Subfamily Members: FGF19 and FGF21.” Journal of Physiology and Biochemistry 75 (2): 229–40.
- Gadaleta, Raffaella Maria, and Antonio Moschetta. 2019. “Metabolic Messengers: Fibroblast Growth Factor 15/19.” Nature Metabolism 1 (6): 588–94.
- Huang, Xinqiang, Chaofeng Yang, Yongde Luo, Chengliu Jin, Fen Wang, and Wallace L. McKeehan. 2007. “FGFR4 Prevents Hyperlipidemia and Insulin Resistance but Underlies High-Fat Diet Induced Fatty Liver.” Diabetes 56 (10): 2501–10.
- Kasperkiewicz, Michael. 2021. “Covid-19, Heat Shock Proteins, and Autoimmune Bullous Diseases: A Potential Link Deserving Further Attention.” Cell Stress & Chaperones.
- Li, Ke, Ling Li, Mengliu Yang, Hua Liu, Guenther Boden, and Gangyi Yang. 2012. “The Effects of Fibroblast Growth Factor-21 Knockdown and over-Expression on Its Signaling Pathway and Glucose-Lipid Metabolism in Vitro.” Molecular and Cellular Endocrinology 348 (1): 21–26.
- Li, Xiaokun, Weiqin Lu, Alexei Kharitonenkov, and Yongde Luo. 2024. “Targeting the FGF19-FGFR4 Pathway for Cholestatic, Metabolic, and Cancerous Diseases.” Journal of Internal Medicine 295 (3): 292–312.
- Yang, Chaofeng, Cong Wang, Min Ye, Chengliu Jin, Weimin He, Fen Wang, Wallace L. McKeehan, and Yongde Luo. 2012. “Control of Lipid Metabolism by Adipocyte FGFR1-Mediated Adipohepatic Communication during Hepatic Stress.” Nutrition & Metabolism 9 (1): 94.
- Zhang, Xizhen, and Wei Yu. 2022. “Heat Shock Proteins and Viral Infection.” Frontiers in Immunology 13 (August): 947789.
- Zhao, Zhuangzhuang, Ling-Dong Xu, Fei Zhang, Qi-Zhang Liang, Yajuan Jiao, Fang-Shu Shi, Biao He, - Pinglong Xu, and Yao-Wei Huang. 2023. “Heat Shock Protein 90 Facilitates SARS-CoV-2 Structural Protein-Mediated Virion Assembly and Promotes Virus-Induced Pyroptosis.” The Journal of Biological Chemistry 299 (5): 104668.

*3. It is intriguing that viral RNAs are not only detected in HEP7 cells. This would suggest that not all hepatocytes respond to viral infection by drastic transcriptome changes, such as HEP2 transitioning to HEP7. Could the authors elaborate on the potential specificities of HEP2 cells that would make them prone to response to viral infection by e.g., increased acute phase response proteins? Similarly, do the authors have a proposed explanation for e.g., the predisposition of HEP4 cells to show signs of activated NFkB and type 1 interferon signaling after viral infection?*

Following the Reviewer’s apt remark, we further enhanced the gene expression and pathway profiles of the SARS-CoV-2+ hepatocyte clusters. Specifically, the HEP2 cluster demonstrated both high metabolic activity and a higher expression of acute phase proteins, which could predispose them to an increased response phenotype. On the other hand, infected cell populations not included in the COVID-19-specific lineage, such as the HEP4 cluster, maintained a high expression of NF-kB, type 1 interferon signaling, and high activation of heat shock proteins. Additionally, these cells presented an elevated autophagy activity by highly expressing crucial genes for the autophagy machinery, including autophagy-related genes (*ATG2B, ATG7, ATG10, ATG5, ATG4C, ATG14*), *MAP1LC3B* which is essential factor for the autophagosome formation, protein kinase AMP (*PRKAA2*), *RPTOR* and *ULK2* that is involved in autophagy initiation. The high abundance of SARS-CoV-2 viral reads in these cells may be attributed to the virus's ability to exploit autophagy pathways (Choi, Bowman, and Jung 2018). By modulating autophagic processes, viruses can prevent the degradation of viral components, thereby enhancing their replication within host cells (Shan et al. 2023).

According to these observations, the following text and supplementary figure have been added to the manuscript:

[**Results**]: “HEP4 cluster presented high expression of NF-kB, type 1 interferon signaling, and heat shock proteins, as well as an elevated autophagy activity. HEP4 cells highly expressed autophagy-related pathways and genes, such as *ATG2B, ATG7, ATG10, ATG5, ATG4C, ATG14*, and *MAP1LC3B,* which is an essential factor for the autophagosome formation, protein kinase AMP (*PRKAA2*), *RPTOR,* and *ULK2* which is involved in autophagy initiation (**Fig. 3b)**. COVID-19-specific lineage clusters, i.e., HEP2, HEP6, and HEP7, were characterized by high expression of acute phase protein genes (*SERPINA1, FGA, FGB, FGG, HP, SAA1, CRP, FTH1, C3,* **Fig 3b, Additional file 2: Fig. S2a**) and by an upregulation of the unfolded protein response pathway (**Additional file 2: Fig. S3a**), which may predispose them to an increased response to viral infection phenotype. On the other hand, we observe an increase also in HEP2 cells which could represent a dynamic response to maintain liver function [3].”

[**Discussion**]: “Terminally differentiated hepatocytes demonstrated both high metabolic activity and an increased inflammatory response, characterized by high expression of acute phase proteins, which may enable the increased response to viral infection pathway activity observed in SARS-CoV-2+ cells of these populations[1]. Additionally, the increased abundance of these cells in COVID-19 patients, along with their elevated typical metabolic processes, may reflect a compensatory mechanism to maintain liver function and address the increased metabolic demands and detoxification needs during disease progression [2]. On the other hand, viral-infected hepatocytes not included in the COVID-19-specific lineage maintained a highly stressed state and elevated autophagy activity. These populations presented high expression and activity of autophagy-related pathways and genes, which could represent an effort of the cells to induce clearance of viruses through viral component encapsulation in autophagosomes, and lysosomal degradation. However, coronaviruses have been shown to turn autophagy into a double-edged sword, and through its modulation, they can prevent degradation, further enhancing their replication and persistence within host cells. [3] [4]. “

**References**

Choi, Younho, James W. Bowman, and Jae U. Jung. 2018. “Autophagy during Viral Infection - a Double-Edged Sword.” Nature Reviews. Microbiology 16 (6): 341–54.

Shan, Tianjiao, Lan-Ya Li, Jin-Ming Yang, and Yan Cheng. 2023. “Role and Clinical Implication of Autophagy in COVID-19.” Virology Journal 20 (1): 125.

**Figure 3b:** Heatmap capturing the expression of marker genes across the hepatocyte and the biliary epithelial cell compartments.

**Additional file 2: Fig. S2a**: Heatmap capturing highly active pathways based on pathway activity scores in Hepatocytes.

*4. Considering the relative small tissue areas that were analyzed in all samples, the authors might as well discuss some limitations that could be due to sampling artifacts.*

We agree with the Reviewer’s comment. The following text has been added to the Discussion section of the manuscript.

[**Discussion**]: “Furthermore, it is important to note that all analyses were performed in a limited section of the patient livers due to the organ’s extensive size and the limitations of current single cell and spatial tissue profiling technologies. However, the amount of tissue sampled in our study via 13G core biopsy is comparable to or greater than a standard 1.5mm liver needle biopsy used in most studies of human liver pathophysiology.”

*5. The manuscript conclusion states "Our study revealed SARS-CoV-2 RNA positive hepatocytes with an expression phenotype similar to infected lung epithelial cells", whereas the cover letter states "We identified human hepatocytes infected by SARS-CoV-2 and characterized their expression profiles at single cell resolution, which are in many ways distinct from infected lung epithelial cells, reflecting the combined viral and systemic effects of COVID-19 on hepatocyte populations.". May the authors clarify these? Furthermore, this point is not explained enough in the manuscript. What would be the distinct/resembling aspects between infected lung and hepatocytes?*

We would like to thank the Reviewer for this comment, since we believe that it would be of interest to the Readers.

In our previous study, focusing majorly on the COVID-19 lung cellular phenotypes (Delorey et al. 2021) lung epithelial cells positive for SARS-CoV-2 viral reads were significantly enriched for TNF, AP1, chemokine and cytokine signaling, as well as SARS-CoV-2 driven cellular responses *in vitro* (Blanco-Melo et al. 2020) and keratinization pathways that may reflect an injury response.

Similarly and strikingly, the SARS- CoV-2 RNA+ hepatocytes were also highly enriched in TNF and SARS-CoV-2 driven cell responses in vitro gene sets (Blanco-Melo et al. 2020) (FDR < 0.01). However, even though the AP1 signaling pathway was higher in the SARS- CoV-2 RNA+ hepatocytes, this difference was not identified as significant following multiple comparison correction (FDR = 0.3177).

On the other hand, despite these similarities, hepatocytes also presented a distinct response phenotype. Unlike the infected lung epithelial cells, interleukin-10 signaling was not identified as increased while interleukin-1, had a significantly higher pathway activity score in the SARS-CoV-2 RNA+ hepatocytes, as compared to the uninfected hepatocytes. We also observed immune and viral infection relevant pathways with significantly higher pathway activity scores in the infected hepatocytes (Figure 1).

The significant pathway scores also highlight liver-specific responses in the infected hepatocytes. MYC targets and oxidative phosphorylation are significantly up-regulated in the infected hepatocytes. MYC is involved in hepatocellular proliferation (Qu et al. 2014) , and overexpression in hepatocytes leads to increased liver fibrosis (Nevzorova et al. 2013) . Regulation of oxidative phosphorylation in hepatocytes is crucial for suppressing inflammation and proliferation (Fu et al. 2013; Santacatterina et al. 2016; Bai et al. 2021). Pathways related to bile acids were identified as significantly down-regulated in infected hepatocytes. Secondary bile acids play a role in modulating inflammatory responses (Buchynskyi et al. 2023).

**Figure 1**: Heatmap capturing pathways based on pathway activity scores in SARS-CoV-2+ hepatocytes compared to SARS-CoV-2- hepatocytes with significant differences (FDR < 0.05).

The following text and the relevant figure (**Additional file 2: Fig. S3g**) have been added to the Results section

**[Results]:** “Pathway activity comparisons between SARS-CoV-2 positive and negative hepatocytes (**Additional file 2: Fig. S3g**) revealed a significantly increased activity in SARS-CoV-2 positive cells of the TNF pathway, which was also identified as enriched in infected lung epithelial cells in Delorey et al. [7]. However, despite these similarities, infected hepatocytes showed increased activity of the IL-1 pathway, which was not observed in the lung single cell atlas, as well as increased activity of the MYC and oxidative phosphorylation pathways. MYC is involved in hepatocellular proliferation [34], and overexpression in hepatocytes leads to increased liver fibrosis [35]. Regulation of oxidative phosphorylation in hepatocytes is crucial for suppressing inflammation and proliferation [36–38]. Pathways related to bile acids were identified as significantly down-regulated in infected hepatocytes. Secondary bile acids play a role in modulating inflammatory responses [39].”

**References**

- Bai, Xiaojie, Yilie Liao, Fangfang Sun, Xia Xiao, and Suneng Fu. 2021. “Diurnal Regulation of Oxidative Phosphorylation Restricts Hepatocyte Proliferation and Inflammation.” Cell Reports 36 (10): 109659.
- Blanco-Melo, Daniel, Benjamin E. Nilsson-Payant, Wen-Chun Liu, Skyler Uhl, Daisy Hoagland, Rasmus Møller, Tristan X. Jordan, et al. 2020. “Imbalanced Host Response to SARS-CoV-2 Drives Development of COVID-19.” Cell 181 (5): 1036–45.e9.
- Buchynskyi, Mykhailo, Iryna Kamyshna, Valentyn Oksenych, Nataliia Zavidniuk, and Aleksandr Kamyshnyi. 2023. “The Intersection of COVID-19 and Metabolic-Associated Fatty Liver Disease: An Overview of the Current Evidence.” Viruses 15 (5). https://doi.org/10.3390/v15051072.
- Delorey, Toni M., Carly G. K. Ziegler, Graham Heimberg, Rachelly Normand, Yiming Yang, Åsa Segerstolpe, Domenic Abbondanza, et al. 2021. “COVID-19 Tissue Atlases Reveal SARS-CoV-2 Pathology and Cellular Targets.” Nature 595 (7865): 107–13.
- Fu, Dong, Kasturi Mitra, Prabuddha Sengupta, Michal Jarnik, Jennifer Lippincott-Schwartz, and Irwin M. Arias. 2013. “Coordinated Elevation of Mitochondrial Oxidative Phosphorylation and Autophagy Help Drive Hepatocyte Polarization.” Proceedings of the National Academy of Sciences of the United States of America 110 (18): 7288–93.
- Muus, Christoph, Malte D. Luecken, Gökcen Eraslan, Lisa Sikkema, Avinash Waghray, Graham Heimberg, Yoshihiko Kobayashi, et al. 2021. “Single-Cell Meta-Analysis of SARS-CoV-2 Entry Genes across Tissues and Demographics.” Nature Medicine 27 (3): 546–59.
- Nevzorova, Yulia A., Wei Hu, Francisco J. Cubero, Ute Haas, Julia Freimuth, Frank Tacke, Christian Trautwein, and Christian Liedtke. 2013. “Overexpression of c-Myc in Hepatocytes Promotes Activation of Hepatic Stellate Cells and Facilitates the Onset of Liver Fibrosis.” Biochimica et Biophysica Acta 1832 (10): 1765–75.
- Qu, Aijuan, Changtao Jiang, Yan Cai, Jung-Hwan Kim, Naoki Tanaka, Jerrold M. Ward, Yatrik M. Shah, and Frank J. Gonzalez. 2014. “Role of Myc in Hepatocellular Proliferation and Hepatocarcinogenesis.” Journal of Hepatology 60 (2): 331–38.
- Santacatterina, Fulvio, Laura Sánchez-Cenizo, Laura Formentini, Maysa A. Mobasher, Estela Casas, Carlos B. Rueda, Inmaculada Martínez-Reyes, et al. 2016. “Down-Regulation of Oxidative Phosphorylation in the Liver by Expression of the ATPase Inhibitory Factor 1 Induces a Tumor-Promoter Metabolic State.” Oncotarget 7 (1): 490–508.

*5. Fig. S6B is moderately convincing to make the point of varying degrees of ductular reaction between L1 to L4. Additional fields of view and quantitations would be more appropriate.*

The images provided in the original submission were very carefully selected and were identified as highly representative by the team of domain experts. Following the Reviewer’s comment, we added low magnification, entire core biopsy images of CK19 stains, and additional blow-up images for further illustration (**Additional file 2, Fig. S8a**). We have further refined this point by providing rigorous quantitation of CK19+ cell numbers and ductular structures, respectively (**Additional file 2, Fig. S8b**) in each liver, which clearly show the various degrees of ductular proliferation (up to a 2-fold difference of average duct counts). We hope this new refined dataset fully addresses the Reviewer's concern.

**MINOR COMMENTS**

*1. Page 6 line 19: Protein names should not be italicized.*

The suggested edit has been performed

*2. Page 17 line 48 did you mean "liver fibrosis"?*

The suggested edit has been performed

*3. Ductular reaction and other histopathology findings have been characterized by a pathologist in only 4 liver samples (L1 to L4). Why were those samples chosen for in-depth analysis? Also, livers 5, 12 and 13 are from patients with a high total bilirubin levels in the blood. How could that affect the findings? Could additional information, such as fibrosis and inflammation scores, be provided for all patients?*

Most participating centers collected only frozen samples, which were used in the single-cell studies. L1-L4 were collected at the beginning of the study, and the protocol included the collection of an additional biopsy, which was formalin-fixed and embedded in paraffin. There were no criteria applied for the selection of these subjects apart from the fact that they were the first 4 enrolled subjects for their collecting center, which we could readily access for pathology assessment. In our manuscript, we clearly specified that these 4 livers represent 4 subsequent cases collected in one of the participating centers (BIDMC). Based on demographics and clinical course, they do not present outlier cases in any specific way. Furthermore, all 4 samples were collected with less than 2h warm ischemia time, which makes them of high technical quality. Unfortunately, there are no additional samples available for the remaining 13 patients that could be utilized to generate similar stains or fibrosis and inflammation scores.

Regarding the increased bilirubin values, we do not believe that it affects the results but that it is one more manifestation of the subclinical COVID-19 secondary cholangiopathy and cholangiocyte remodeling observed in our single cell studies. COVID-19 cholangiopathy is also known to be associated with increased LFTs, including bilirubin (Rasheed et al. 2023). We believe our findings on ductular reaction and fibrotic response, which were also observed in the in-depth histology studies, are consistent with this observation.

**References**

- Rasheed, Mazen Abdalla, Vinícius Remus Ballotin, Lucas Goldmann Bigarella, and Jonathan Soldera. 2023. “Post-COVID-19 Cholangiopathy: Systematic Review.” World Journal of Methodology 13 (4): 296–322.
